# Identification of Bacteria and Viruses by Pyrolysis–Gas Chromatography–Ion Mobility Spectrometry

**DOI:** 10.1021/acs.analchem.6c04057

**Published:** 2026-09-02

**Authors:** Daniel Röckrath, Moritz Hitzemann, Alejandra Vargas Valderrama, Arne Hoehne, Maryam Damen, Georgios Kirtsanis, Georgios Dolias, Martin Lippmann, Ayke Eckhardt, Christian Thoben, Guillaume Lepage, Stefan Zimmermann

**Affiliations:** † Institute of Electrical Engineering and Measurement Technology, 26555 Leibniz University Hannover, Appelstr. 9A, 30167 Hannover, Germany; ‡ Belgian Defence Laboratories (DLD), Quartier Major Housiau, Martelarenstraat 181, 1180 Vilvoorde, Belgium; § Royal Military Academy (RMA), Avenue de la Renaissance 30, 1000 Brussels, Belgium; ∥ Information Technologies Institute (ITI), Centre for Research and Technology Helias (CERTH), 6th Km Charilaou - Thermis Rd, 57001 Thermi, Thessaloniki, Greece

## Abstract

Rapid and reliable detection of biological agents is crucial in both military and civilian contexts. Here, we present a promising field-deployable approach that combines a simple, self-built pyrolyzer (Py) with a gas chromatograph (GC) and an ultra-fast polarity switching ion mobility spectrometer (IMS). The Py–GC–IMS provides a sensitive method for detecting viruses and bacterial pathogens in liquid and solid samples. During pyrolysis, the sample is rapidly heated under an inert gas atmosphere in the absence of oxygen, leading to a transition of the sample from its nonvolatile solid or liquid state to the gas phase by breaking up the sample into its volatile constituents. Pyrolysis is followed by gas chromatography, separating the volatiles in a first dimension (retention time). For highly sensitive detection of the volatiles eluting from the GC, an ultra-fast polarity-switching IMS is used that also provides a second dimension of separation (drift time). To demonstrate the potential of this hyphenated device, five viruses and five bacteria, including two strains of the same bacterium, and simulants of biological warfare agents, were analyzed. Additionally, an unsupervised machine learning approach was applied to a representative subset of the available classes to showcase the potential of combining the device with state-of-the-art machine learning methods. So far, identification of bacteria at concentrations in the range of 10^8^ colony-forming units per microliter (cfu/μL) and viruses at concentrations in the range of 10^6^ viral particles/μL and lower is possible either by the GC–IMS fingerprints or by the unsupervised machine learning methods used.

## Graphical abstract

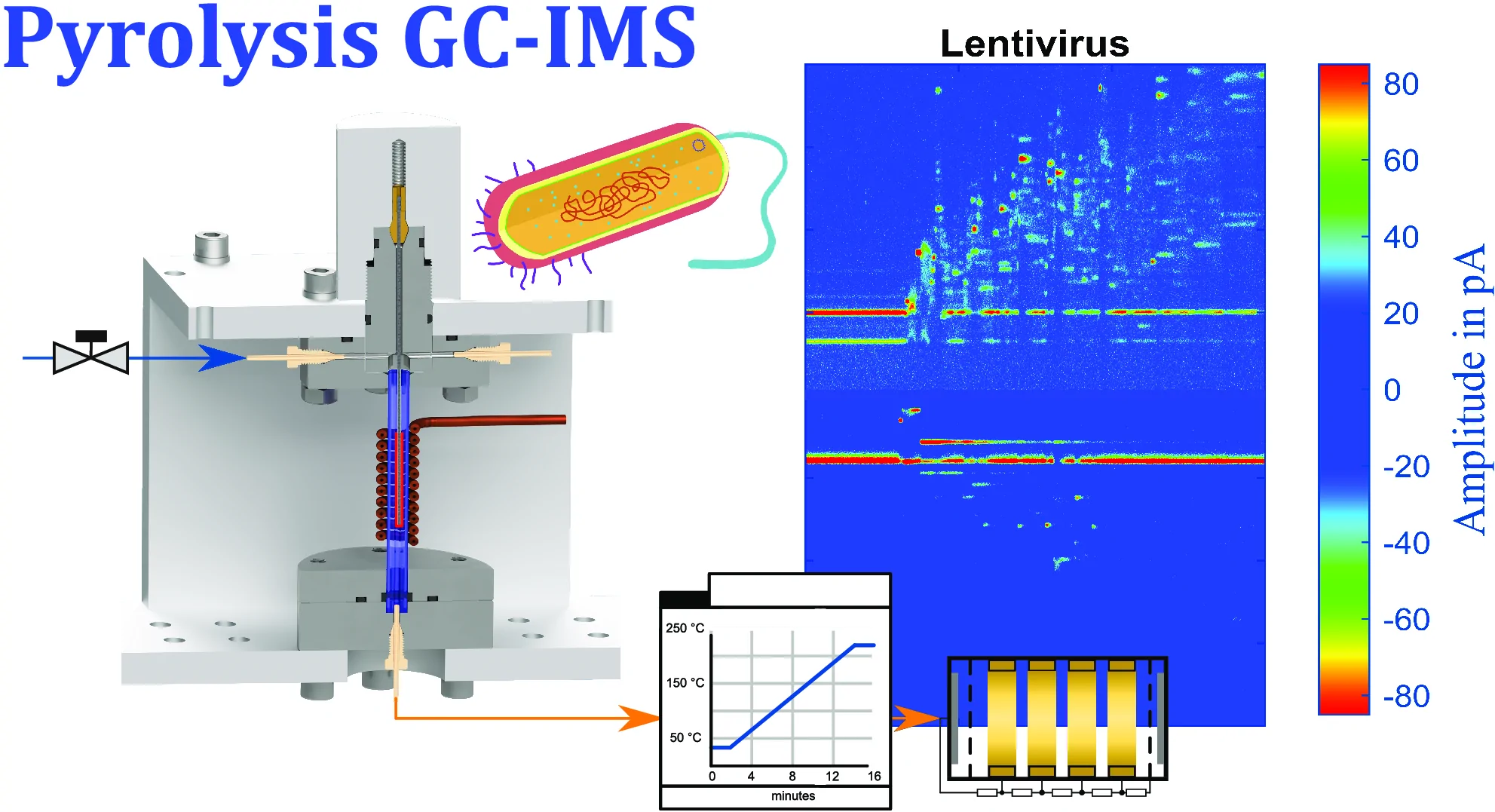

## Introduction

In the current socio-political context, the risk of both naturally occurring and deliberately released biological (BWAs) and chemical warfare agents (CWAs) continues to increase.^1,2^ Historically, detection technologies for biological and chemical threats have evolved independently due to their different physicochemical properties, yet a unified solution could enable rapid on-site threat assessment. BWA detection relies primarily on biomolecular methods such as polymerase chain reaction (PCR), targeting nucleic acids, and therefore is not applicable to chemical agents.^3–5^ Conversely, CWAs are typically identified based on their volatile chemical signatures using mass spectrometry (MS),^6–8^ IMS,^9–12^ gas chromatography–ion mobility spectrometry (GC–IMS),^13^ and gas chromatography–mass spectrometry (GC–MS).^14,15^ Although these analytical platforms could, in principle, be adapted for biological materials, such as matrix-assisted laser desorption/ionization time-of-flight (MALDI-TOF),^16–18^ two major limitations exist. First, biological samples are intrinsically not volatile and often present in liquid or solid form, requiring conversion into the gas phase prior to GC separation. Second, microorganisms are composed of diverse macromolecules that generate highly complex chromatograms, making interpretation computationally demanding.

Analytical pyrolysis offers a promising approach to overcome these limitations.^19–22^ The term pyrolysis describes the process of degradation of molecules in the absence of oxygen using thermal energy. The degradation processes can occur in different ways, depending on the molecule, leading to the breaking of chemical bonds and the production of free radicals.^20^ The pyrolysis products that are generated may subsequently enter the gas phase, thereby providing information about the original biological material. This can form a quite complex chromatogram requiring 2D analytical information. Several studies coupling pyrolyzers to GC–MS have demonstrated that pyrolysis efficiently degrades and volatilizes fatty acids, particularly those originating from bacterial membranes.^21,23–25^ These lipid-derived fragments serve as robust biomarkers for bacterial identification, and pyrolysis circumvents the need for labor-intensive classical lipid extraction procedures. Related thermochemolysis approaches have shown that other low-volatility biomolecules, such as carbohydrates from bacterial peptidoglycans or dipicolinic acid, a hallmark of bacterial endospores, can also be converted into volatile derivatives suitable for GC–MS analysis.^26,27^ Using these methods, endospores from several *Bacillus* species, including those closely related to *Bacillus anthracis*, have been successfully differentiated.^24^ Recently, we demonstrated that pyrolysis–gas chromatograph–ion mobility spectrometry (Py–GC–IMS) can reliably identify anaerobic bacteria based on their pyrolysis-derived chemical signatures. These results highlight the potential of pyrolysis-enabled GC platforms to expand the range of detectable microorganisms and suggest that the approach could be extended to other biological agents, including viruses, whose structural components may also yield characteristic pyrolysis fragments.^22^ Additionally, Py–GC–IMS promises field-deployability with lower measurement times than PCR^28^ and no vacuum requirements like MALDI-TOF.^29^

Several heating strategies exist for achieving the temperatures required for pyrolysis, including resistive heating, pyrolysis furnaces, and inductive heating using Curie point materials.^20^ Resistive heating uses a single wire for rapid heating through direct contact with a power source. Unfortunately, it requires precise control and temperature measurement. On the other hand, a pyrolysis furnace has large thermal capacity, making it difficult to achieve short temperature rise times. Additionally, it has higher complexity for temperature control due to unknown sample temperatures.^20^ In contrast, the Curie point method uses a high-frequency induction coil to generate a magnetic field, heating a ferromagnetic sample holder through hysteresis and eddy current losses. The induction frequency is typically in a range from a couple of 100 kHz up to 1 MHz, depending on the used induction coil, the used ferromagnetic material, and the dimensions of the ferromagnetic sample holder. Above the Curie temperature, ferromagnetic properties and hysteresis losses diminish due to the material changing to paramagnetic properties, self-regulating to the material-specific Curie temperature.^30^ Due to this effect, the ferromagnetic sample holder temperature is self-regulating without external measurement and control electronics, but is limited to the specific Curie temperature. This approach provides high reproducibility due to the reliance on a physical effect and the high heating rates that can be achieved due to the low thermal capacity of the ferromagnetic sample holder.

The magnetic field for inductive heating is generated by a specially developed full-bridge inverter in combination with a copper coil optimized for temperature control.^31^ Key parameters, including the GC inlet split ratio and heating rate, were optimized for biological sample analysis.

Traditionally, the complexity of pyrolysis-based chromatograms has been addressed by selecting specific biomarkers for microbial identification. However, recent advances in machine-learning (ML) and deep-learning (DL) algorithms have demonstrated strong potential for classifying microorganisms directly from high-dimensional spectral data.^32–34^ Building on these developments, the present study analyzes five viruses and five bacteria, including BWA simulants, using Py–GC–IMS combined with unsupervised ML analysis. Comparison of their 2D-chromatograms revealed reproducible chemical fingerprints that enabled classification according to major structural features, such as Gram-positive versus Gram-negative cell envelopes in bacteria, and the presence or absence of a lipid envelope in viruses. ML-based analysis further strengthened species-level differentiation. Together, these results demonstrate that Py–GC–IMS combined with ML algorithms can detect and differentiate multiple biological agents, demonstrating its potential as a rapid, extraction-free approach for microbial identification.

## Experimental Section

The Curie temperature *T*_C_ is defined as the temperature at which the ferromagnetic properties of a material disappear. It is a material or substance-specific constant. At temperatures above the Curie temperature, the oscillation of the atoms in the lattice increases significantly, and the coupling forces are overcome. As a result, the magnetic moments are randomly arranged, and constructive superposition is not possible, leading to a net magnetization of zero. Above the Curie temperature, ferromagnetic materials enter the paramagnetic state. If the temperature falls below the Curie point, the material becomes ferromagnetic again. Regardless of whether the material was previously magnetized, the net magnetization is zero. A new magnetization can only be generated by an external magnetic field. By combining different ferromagnetic materials, the Fe50Ni50 wire used here has a Curie temperature of 520 °C.^34,35^ This temperature is often used in other publications related to Py–GC–MS or pyrolysis in general.^35,36^

Since the Curie point method is self-regulating, a direct temperature measurement is not necessary for the device. However, for validation purposes and to determine the heating ramp, the temperature of the ferromagnetic sample holder is measured using an infrared thermometer from Optris (CTlaser 3MH1 with CF2 lens giving a measurement spot of ≤ 0.5 mm and a fast sampling rate of 1 kHz). This optical infrared thermometer can measure the temperature of a metal object through glass. To measure the temperature of the ferromagnetic sample holder, the induction coil requires a small gap, as illustrated in Figure 1a. Due to this gap, there may be a magnetic field inhomogeneity inside the induction coil. To estimate such a field inhomogeneity, we performed simulations in CST Studio, and the results are shown in Figure 1b. The current through the induction coil was set to 10 A, corresponding to the current in the physical setup. The simulation results suggest that the gap has only little impact on the global magnetic field distribution within the coil. In particular, no substantial reduction in field strength was observed in the region of the gap compared to other parts of the coil. This suggests that the overall field homogeneity is only marginally influenced by the gap. Based on these findings, it can be assumed that the thermal behavior of the ferromagnetic sample holder in a coil with such a gap is similar to that in a fully enclosed coil, as used in the final pyrolysis unit. However, local deviations cannot be entirely excluded. It should be emphasized that the simulations are intended to provide a qualitative assessment of the gap’s influence on the field distribution. A precise quantitative description is limited by the omission of parasitic effects, potential disturbances, and simplified material properties. The physical induction coil consists of 15.5 turns, has an inner diameter of 10 mm, and a length of approximately 53 mm. Its inductance is 600 nH, and its ohmic resistance is 3.6 mΩ. The voltage is applied to the coil with a full-bridge inverter with an external frequency generator.^31^

**Figure 1:**
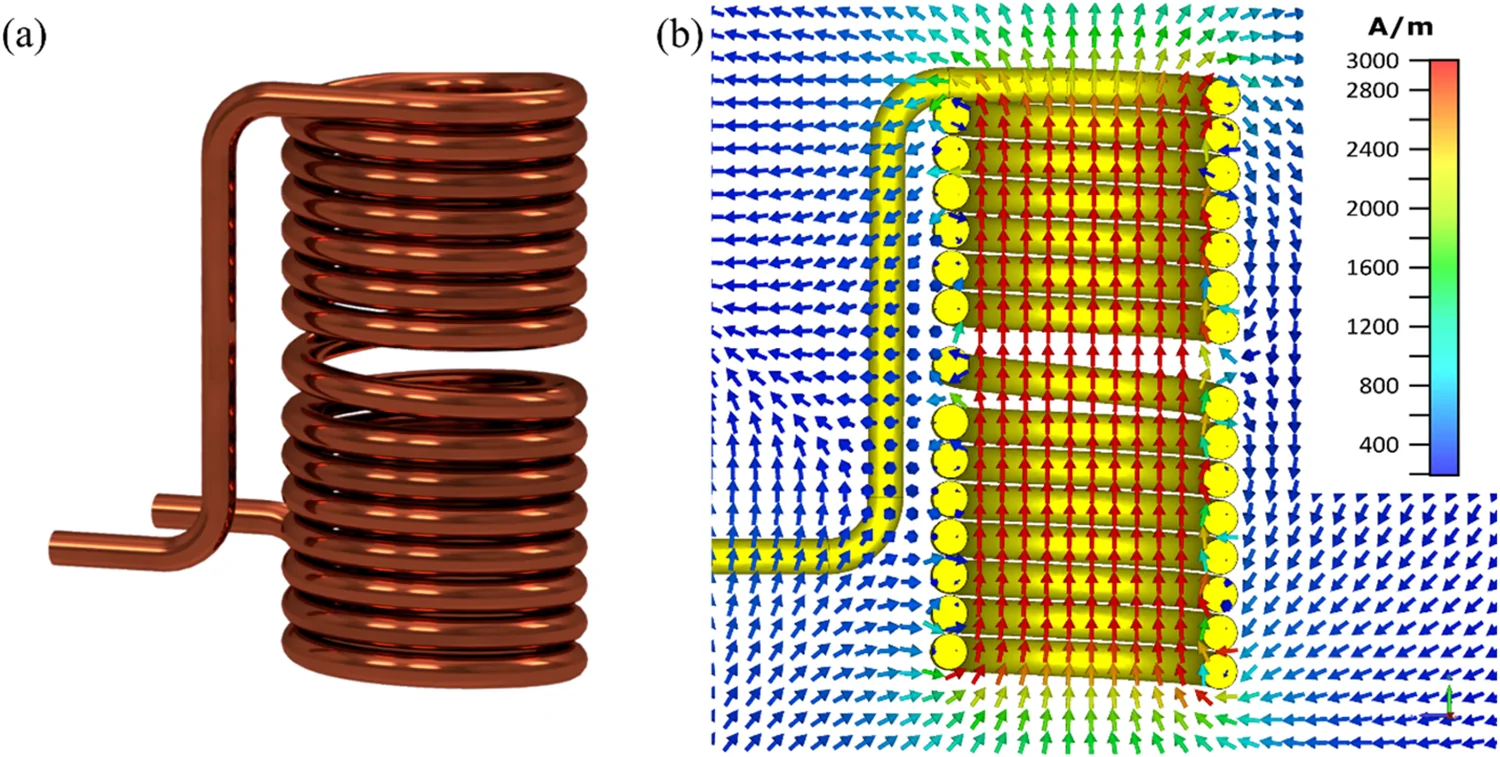
CAD model of the used induction coil with a gap between two windings (a) and the corresponding CST simulation of the magnetic field strength at a current of 10 A (b).

The low-cost, self-built pyrolysis unit is described in detail by Lippmann et al., including assembly instructions.^31^ To guarantee that the sample is pyrolyzed in an oxygen-free atmosphere, the ferromagnetic sample holder is mounted inside a glass liner (Agilent, 5190–2292) and flushed with nitrogen. The glass liner is surrounded by the 1 mm copper induction coil. The mechanical construction is shown in the Supporting Information in Figure S1 and consists of two parts. Figure S1a shows the handle for the ferromagnetic sample holder, which is designed for the holder to be inserted from below and automatically locked in place by the chuck spring combination. The lower part of the main body features two O-rings for sealing; the lower O-ring seals the handle to the pyrolysis chamber, as shown in Figure S1b. The handle and the glass liner are connected gas-tightly with an O-ring in the outer adapter. An additional O-ring located on the bottom of the pyrolysis chamber seals the glass liner with the outlet for connection to, e.g., a GC. By changing the supplied power to the induction coil, the temperature rise time can be controlled.

For the measurements of the different biological samples, this low-cost pyrolyzer is coupled to a commercial GC (Agilent 7890A) hyphenated with an ultra-fast polarity switching IMS as the GC-detector. The combined setup is shown in Figure 2 with detailed gas flows for the nitrogen, sample, and waste gas. Dried, clean nitrogen with 5 mL/min is used as carrier gas for the GC column. The GC column is a Restek Rxi-5Sil MS 30 m (inner diameter 530 μm, film thickness 1.5 μm). The low-cost pyrolyzer is connected via a heated transfer line (100 °C) to the heated GC multimode inlet (250 °C) with a split ratio of 1:10. GC separation was carried out with the following temperature profile. The start temperature was 40 °C for 2 min after injection, then the temperature was increased to 220 °C at a rate of 15 K/min and then kept at 220 °C for an additional 2 min for a total GC runtime of 16 min. The final 6 min of the GC run at high temperatures are used to clean any unwanted pyrolyzed fragments from the column. The cooling until the GC reaches 40.0 °C again is 5 min ± 10 s, depending on the ambient temperature. The GC has a retention time repeatability of <0.008%.^37^ The GC method was used previously with the same device in Hitzemann et al., resulting in good reproducibility.^38^ Simultaneous measurement of both polarities with the IMS allows detection of fragments of either polarity during one single GC run. This could lead to the detection of more diverse fingerprints since both polarities contain information. The ultra-fast polarity switching IMS is made out of printed circuit boards with a field-switching ion shutter. The specific operating parameters of the ultra-fast polarity switching IMS are given in Table 1. The GC–IMS coupling is the same as described in detail by Hitzemann et al.^38^ Data acquisition was performed using the measurement software published by Kobelt et al.^39^

**Figure 2:**
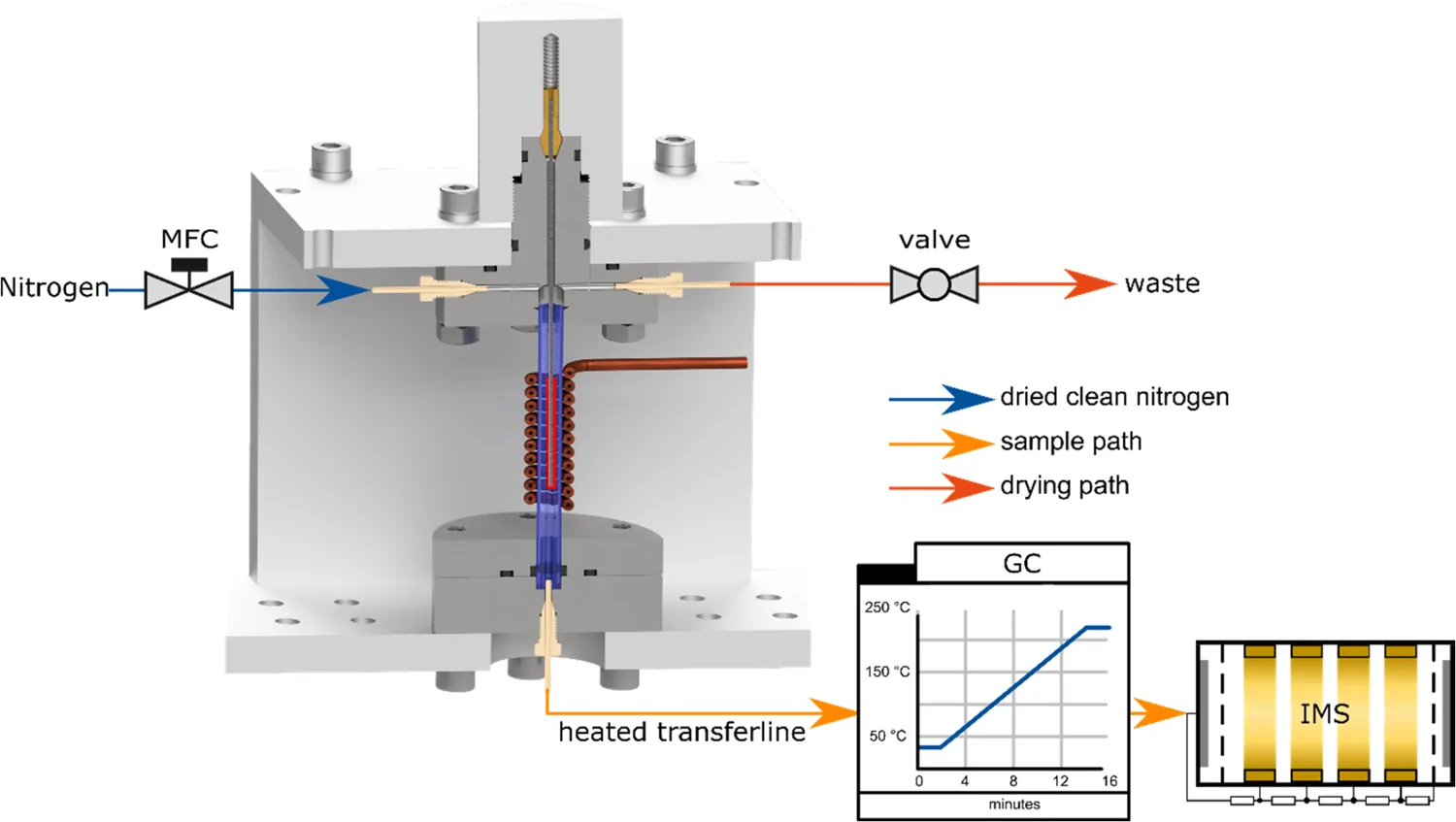
Py–GC–IMS system with heated transfer lines in between. The GC features a temperature ramp from 40 to 220 °C.

**Table 1:** Operating Parameters of the Ultra-Fast Polarity Switching IMS

parameter	Value
drift length	103 mm
drift region cross section	20 mm × 20 mm
source activity (tritium)	70 MBq
drift voltage	±8 kV
aperture voltage	±220 V
injection voltage	±600 V
blocking voltage	±100 mV
repetition rate (per spectrum)	40 Hz
injection pulse width	200 μs
IMS pressure	1008 hPa
IMS temperature	28 °C
dew point of the drift and sample gas	–90 °C (90 ppb_v_ water vapor concentration)
drift gas flow (purified air)	150 mLs/min^a^
sample gas flow with GC (purified nitrogen)	5 mLs/min

a mLs/min: milliliter standard per minute, mass flow at reference conditions 20 °C and 1013.25 hPa.

To demonstrate the capability of pyrolysis coupled to GC–IMS and to differentiate biological samples, a set of bacteria and viruses was analyzed. Bacteria and viruses were chosen to represent structural diversity, including Gram-positive and Gram-negative species and enveloped and nonenveloped types of viruses. This list also includes relevant microorganisms, including model organisms and surrogates of human pathogens (*Escherichia coli*, lentiviral vector­(LVC)) and/or of BWAs such as *Bacillus cereus* and *Bacillus atrophaeus* for *Bacillus anthracis*, *Yersinia enterocolitica* for *Yersinia pestis*, or *Francisella philomiragia* for *Francisella tularensis* (see Table S1). All of these were measured in three concentrations, which were diluted with endotoxin-free ultra-pure water (TMS-011-A, Sigma-Aldrich), and in the case of the bacteria, also from three different colonies. The measured fatty acids were also ordered from Sigma-Aldrich, namely propionic acid (94425–1 ML-F), butyric acid (8004570100), and pentanoic acid (75054–5 ML).

## Sample Preparation

Three Gram-negative strains, *E. coli* (DSM 500), *F. philomiragia* (DSM 7535), and *Y. enterocolitica* *subsp. Enterocolitica* (DSM 4780) and three Gram-positive strains, *B. atrophaeus* (DSM 675) and *B. cereus* (DSM 2302 and ATCC 14579) were recovered from frozen glycerol stocks by streaking on tryptic soy agar plates (TSA; VWR Chemicals, Belgium) and incubating overnight either at 37 °C (*E. coli*, *F. philomiragia*), or 30 °C (*Y. enterocolitica*, *B. atrophaeus*, *B. cereus*). Individual colonies of each strain were inoculated in 30 mL of tryptic soy broth (TSB; VWR Chemicals, Belgium) and cultured under agitation at 180 rpm for 16 h at 37 °C (30 °C for *B. atrophaeus* *)*. For enumeration, 500 μL of each overnight culture was serially diluted (10^–8^ – 10^–10^) in TSB, and 100 μL of each dilution was plated on TSA to estimate colony-forming units (CFU). The remaining 29.5 mL of culture was harvested by centrifugation at 4000 x g for 20 min at 4 °C. The bacterial pellet was washed once with 10 mL of sterile phosphate-buffered saline (PBS; Cytiva, USA). Cells were inactivated by heating at 95 °C for 1 h in a thermoshaker at 500 rpm. To confirm complete inactivation, 100 μL of the treated suspension was seeded on TSA and incubated under the appropriate growth conditions; colonies were not observed after 20 days. Inactivated cells were collected by centrifugation (4000 x g, 20 min, 4 °C), washed twice with 10 mL of ultra-pure water, and the resulting pellet was stored at 4 °C until further analysis.

LVC (VB010000-9492agg), Murine Leukemia Virus (MMLV) (VB010000-9307ddn), Baculovirus (VB010000-9305vtu), and Adeno-associated Virus (AAV) (VB010000-9278ffw) were purchased from VectorBuilder (USA). Bacteriophage MS2 was amplified from a previous stock using *E. coli* K-12 MC4100 pOX38. The culture was centrifuged to pellet the host bacteria (4000 x g, 20 min, 4 °C) and filtered through a 0.45 μm filter to remove any remaining host cells. All viruses were further filtered using 10 kDa Vivaspin 500 centrifugal concentrators (Merck, Belgium) according to the manufacturer’s instructions and resuspended in H_2_O. Samples were heat-inactivated at 75 °C for 30 min and stored at −20 °C until analysis.

## Sample Analysis by Py–GC–IMS

The biological samples were prepared by diluting them with lipid-free ultra-pure water (TMS-011-A). Bacterial samples were resuspended at a concentration of 4 × 10^8^ cfu/μL. This concentration was selected because it produced well-defined peaks in the 2D-chromatogram, with inverse reduced ion mobility plotted against retention time, as observed in the initial tests. A concentration of 4 × 10^8^ cfu/μL was chosen as the starting point, and variations in peak number and intensity were achieved by diluting this concentration to half or a quarter of 4 × 10^8^ cfu/μL, as visible in Figure S6. For the viruses, this value varied because the supplied concentrations were lower than those of the bacterial samples (see Table S1). In addition, the viral concentrations provided by the suppliers were reported in different, non-equivalent units depending on the viral strain (plaque-forming units (pfu), transducing units (tu), or genome copies (gc) per μL). Therefore, throughout the manuscript, they are referred to as viral particles/μL. The highest viral concentration tested was 2 × 10^9^ viral particles/μL, while the lowest was 5 × 10^3^ viral particles/μL. Thus, the initial concentrations were used to obtain visible information in the 2D-chromatogram. Each bacterial sample was placed in a weighed glass vial and then filled with lipid-free ultra-pure water to achieve a concentration of 4 × 10^8^ cfu/μL. Each was stored in a new vial containing a total volume of 200 μL, as used for the measurements in Table S2, where the weight includes the vial weight as well as the liquid. For measuring, the ferromagnetic wire was dipped into the vials at their respective concentrations for 3 s, after which the sample adhered to the wire by adhesive forces. The vial was vortexed beforehand to ensure a homogeneous bacterial distribution. The ferromagnetic wire was inserted into the pyrolysis region with a drying step for 5 min. During this time, the wire was flushed with clean, dried nitrogen through the open exhaust valve. This allowed for the residual water to be removed from the ferromagnetic sample holder. After the drying step, the exhaust valve was closed, and the gas flow reached the GC inlet. Blank measurements containing only the clean ferromagnetic wire were performed between each measurement to ensure that no carryover or residues were left.

## Unsupervised Machine Learning Analysis

To showcase the potential of applying complex ML/DL algorithms for the classification of biological samples, we conducted an unsupervised ML analysis of the measurements. For this analysis, we selected one representative class from each parent category, *F. philomiragia*, *B. cereus*, AAV, and LVC, to evaluatethe application of ML algorithms to the proposed data set. Principal component analysis (PCA) and hierarchical cluster analysis (HCA) were applied to the four measurements of each selected class. A conda environment with Python version 3.8.20 was created, and the ML algorithms were trained using scikit-learn version 1.3.2.

## Results and Discussion

The temperature rise time (10% to 90% of end temperature) for the Fe50Ni50 ferromagnetic wire used for the pyrolyzer from ambient temperature to the Curie temperature of 520 °C was 1.37 s at the drive voltage of 55 V, as shown in Figure 3. This shows that the pyrolyzer can heat sufficiently fast, allowing for instant pyrolysis while forming a small volatile sample cloud transferred to the GC. As presented in Lippmann et al., the pyrolyzer heating ramp is highly reproducible in both the ramp itself and the end temperature, being within measurement errors of the used optical temperature sensor.^31^

**Figure 3:**
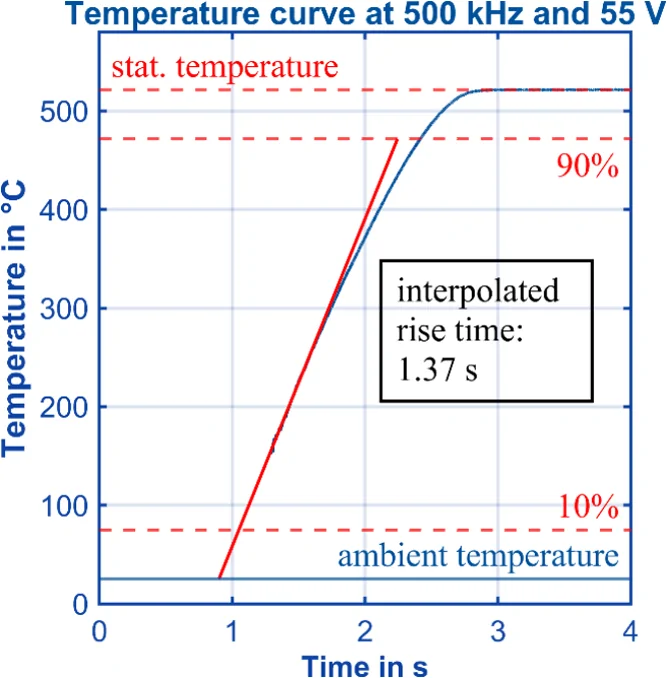
Heating curve of the Fe50Ni50 ferromagnetic wire from the ambient temperature to the Curie temperature of 520 °C.

To investigate the variability of the sample uptake, nine independent wire-loading experiments were performed using an *E. coli* suspension with a concentration of 4.0 × 10^8^ cfu /μL. The results are shown in Table S2. The vials showed an average difference in weight of 303 μg with a 24.25% RSD. This strong variation makes precise quantification difficult. However, as long as enough material is pyrolyzed for a clear fingerprint, detection is possible.

Starting with a measurement solely with the ferromagnetic sample holder shown in Figure S2, no interfering peaks were observed. In the 2D-chromatogram, only the two reactant ion peaks (RIP) are visible (RIP^+^ at 1/*K*_0_ = 0.51 Vs/cm^2^ and RIP^–^ at 1/*K*_0_ = 0.49 Vs/cm^2^). The ion mobility for the negative polarity will be shown as negative values for easier differentiation, even though ion mobility is always positive. The additional peak is correlated to NH_4_^+^ at 1/*K*_0_ = 0.45 Vs/cm^2^.^40,41^ Two further minor peaks (not identified) likely originate from GC residues. This provides a sufficiently clean baseline for the following experiments.

After this, different types of water, including tap water, deionized water (DI-water), and lipid-free ultra-pure water, were tested. The water was used as a solvent for the biological samples. Since tap water contains minerals and may also contain microorganisms, obtaining a clean spectrum might not be possible.^42,43^ DI-water and lipid-free ultra-pure water, considered sterile, contain negligible amounts of residual material, resulting in a reduced or absent background matrix.^44^ With all three different water types shown in Figures S3 to S5, they all show clean, empty 2D-chromatograms. Even though the three chromatograms look clean, the minerals could cluster and thus lead to additional fingerprint signals. Nevertheless, the following biological measurements were conducted using lipid-free ultra-pure water.

Two exemplary 2D-chromatograms are displayed, showing distinct, well-resolved peaks. Figure S6 (2 × 10^8^ cfu/μL) shows the first colony of *E. coli*, and Figure 4 (4 × 10^8^ cfu/μL) corresponds to the second colony with increased sample concentration. As is visible, the spectrum has various peaks in the positive ion mobility and six in the negative ion mobility. For the positive ion mobility, there are often three peaks at about the same retention time with slightly different reduced inverse ion mobilities visible (marked with white circles), for instance, at a retention time of 250 s. These could be a set of monomers and dimers of dienes, alkenes, and alkanes with increasing carbon amounts for each set.^20^

Comparing both 2D-chromatograms clearly shows identical but more intense peaks for the higher concentration, as expected. Further additional peaks appear at the same retention time with a higher inverse reduced ion mobility. These could be explained by clustering, for instance, dimers. The appearance of clustered ions adds additional information to fingerprints, but adds complexity to the interpretation of fingerprints. Thus, a more in-depth analysis might be needed, for instance, peak selection or ML algorithms, as shown later.

**Figure 4:**
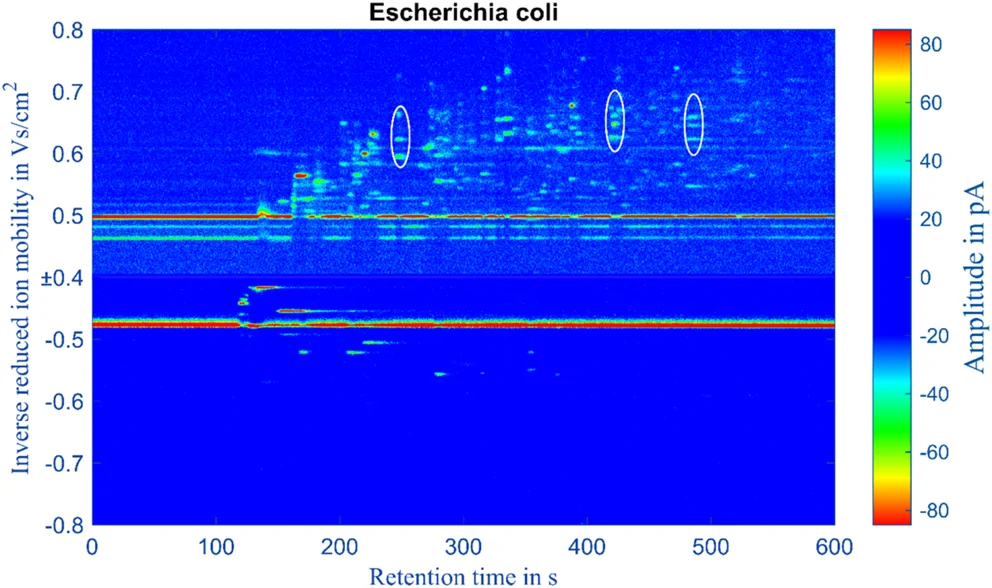
2D-chromatogram of the Gram-negative bacterium *Escherichia coli* measured with the Py–GC–IMS at a concentration of 4 × 10^8^ cfu/μL (1 × 10^8^ cfu).

Figure 5 shows a 2D-chromatogram of the *B. cereus* bacteria. As is directly visible, the total number of peaks is greatly reduced compared to *E. coli*. This reduction is consistent with structural differences in the bacterial cell envelope. Gram-negative bacteria, such as *E. coli*, have an outer membrane rich in lipids, resulting in a higher overalllipid content. Because lipids are among the most abundant molecules in bacterial cells, they contribute strongly to the observed spectral features.^45,46^ In contrast, Gram-positive bacteria, such as *B. cereus*, have a thick peptidoglycan layer with relatively low lipid content.The difference is especially evident at higher inverse reduced ion mobilities and higher retention times, where signals of lipids are expected and where the Gram-positive bacterium shows almost no peaks. Overall, the two bacterial types can be distinguished at first glance.

**Figure 5:**
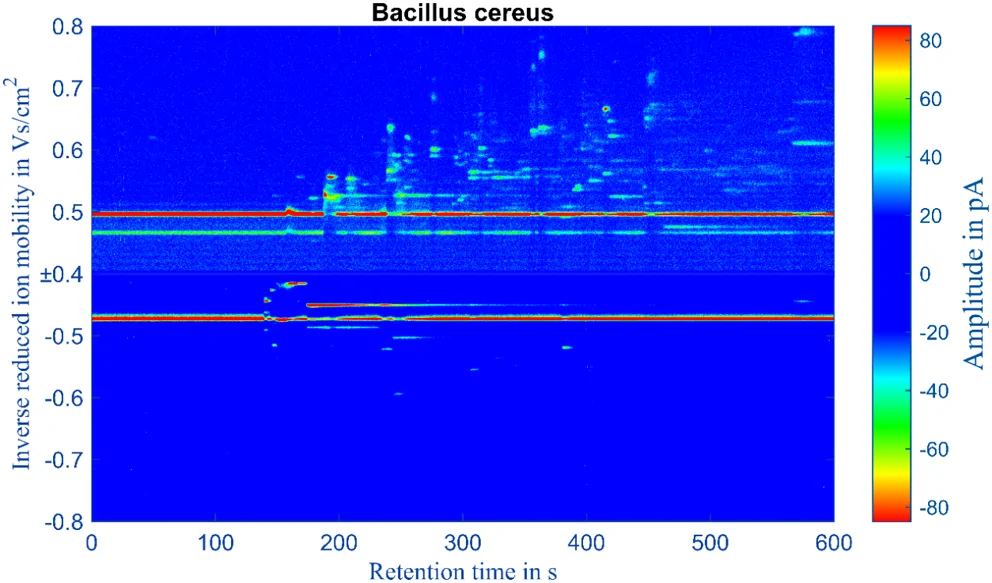
2D-chromatogram of the Gram-positive bacterium *Bacillus cereus* measured with the Py–GC–IMS at a concentration of 4 × 10^8^ cfu/μL (1 × 10^8^ cfu).

Figure 6 shows the 2D-chromatogram of LVC, which is an enveloped virus. It has more negative peaks than the bacteria measured before. The positive polarity also differs from the aforementioned bacteria, with an increased number of peaks, especially at higher inverse reduced ion mobilities and higher retention times. Comparing the LVC to all the measured biological samples highlights its complexity in terms of the total number of peaks across both polarities. This allows easy differentiation of LVC from the other biological samples measured. This may suggest that the LVC pyrolyzes into multiple fragments and that the viral envelope is quite complex. Although it is possible to differentiate LVC from the others at first glance, adding more bacteria and viruses to the data set would possibly require the use of ML at some point to differentiate the fingerprints.

**Figure 6:**
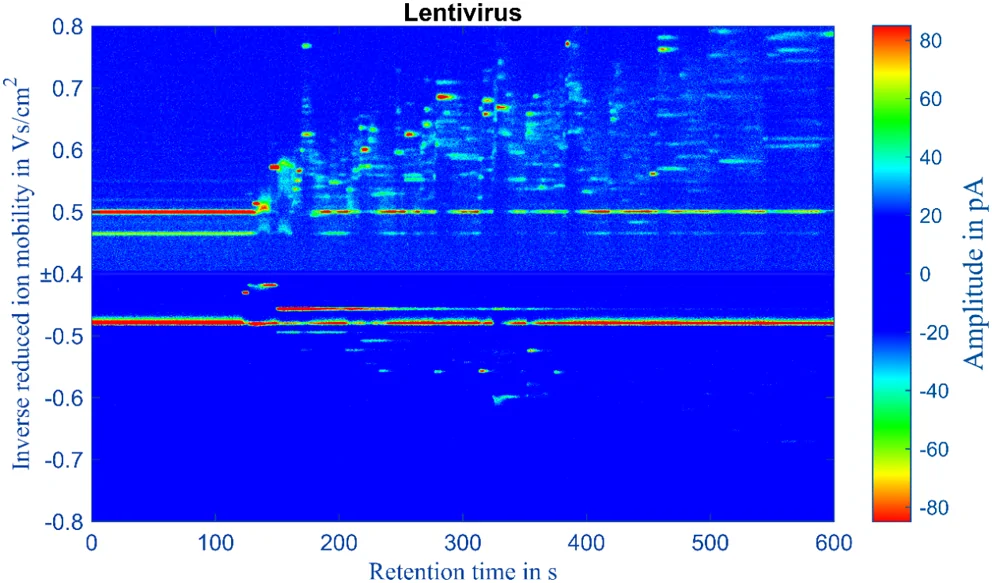
2D-chromatogram of the enveloped virus lentiviral vector measured with the Py–GC–IMS at a concentration of 2 × 10^9^ viral particles/μL (7 × 10^8^ viral particles).

2D-chromatograms of all measured biological samples can be found in Figures S6 to S15, ordered by Gram-negative and Gram-positive bacteria, and nonenveloped and enveloped viruses. The bacteria shown in the main manuscript are also shown at different sample concentrations in the Supporting Information. Most of them can be easily differentiated at first glance. As expected, differences between enveloped and nonenveloped viruses were also observed, with the former showing a more complex spectrum. As for Gram-positive and Gram-negative bacteria, these differences may rely on the presence or absence of a lipid envelope, which introduces additional membrane lipids and thus their fragments that contribute to the increased number of detectable peaks.^45,46^

Distinguishing between the four types of bacteria and viruses is straightforward. Figure 7 shows the fingerprints. The fingerprint features were selected with our own software within MATLAB. Here, we focused on some of the most significant peaks that allow for differentiating all tested biological samples. The criteria were visibility, reproducibility, and intensity for each of the chromatograms. This resulted in 33 significant peaks (features) in positive polarity. As can be seen, the number of peaks varies greatly between the biological samples; for example, LVC has eight peaks not visible in other samples. The dilutions are usually at 4 × 10^8^ viral particles/μL, 2 × 10^8^ viral particles/μL, and 1 × 10^8^ viral particles/μL. As is visible in Figure 7, the peak intensity between each biological sample varies a bit according to the measured concentration and extracted amount of sample.

Some peaks, especially peak 1, are visible in most of the used bacteria and viruses, with the exception of *B. cereus* and BVC, suggesting that this pyrolyzed compound may be a basic structure, possibly part of a general cellular basis or a preparation/culture contaminant. This is the case for a variety of peaks not shown in the comparison. On the other hand, peaks 20 to 28 are exclusive to LVC, except for peak 23, which is solely shared with MMLV. Both of these remain distinct from all other measured biological samples. Most of the peaks are shared by some, but are usually different between biological samples. Overall, the selected fingerprints exhibit a unique peak distribution for each biological sample. A differentiation is therefore achieved by analyzing only the different peaks in the positive polarity, without needing to take a more detailed approach that also analyzes the intensity and integral of each peak or peaks in the negative polarity.

For the negative polarity, the peaks were selected in the same way. Compared to the positive polarity from Figure 7 (a total of 33), the total number of significant peaks in the negative polarity (a total of 16) is lower, as displayed in Figure S16. Interestingly, the negative polarity allows for a distinction of some of the measured biological samples, but due to the reduced total peak number, the distinction is not as accurate as the positive polarity. However, some of the viruses, especially the LVC, show a better distinction that would not be possible with only the positive ion polarity; see Figure S16 with the characteristic peaks 10 and 11 for LVC.

**Figure 7:**
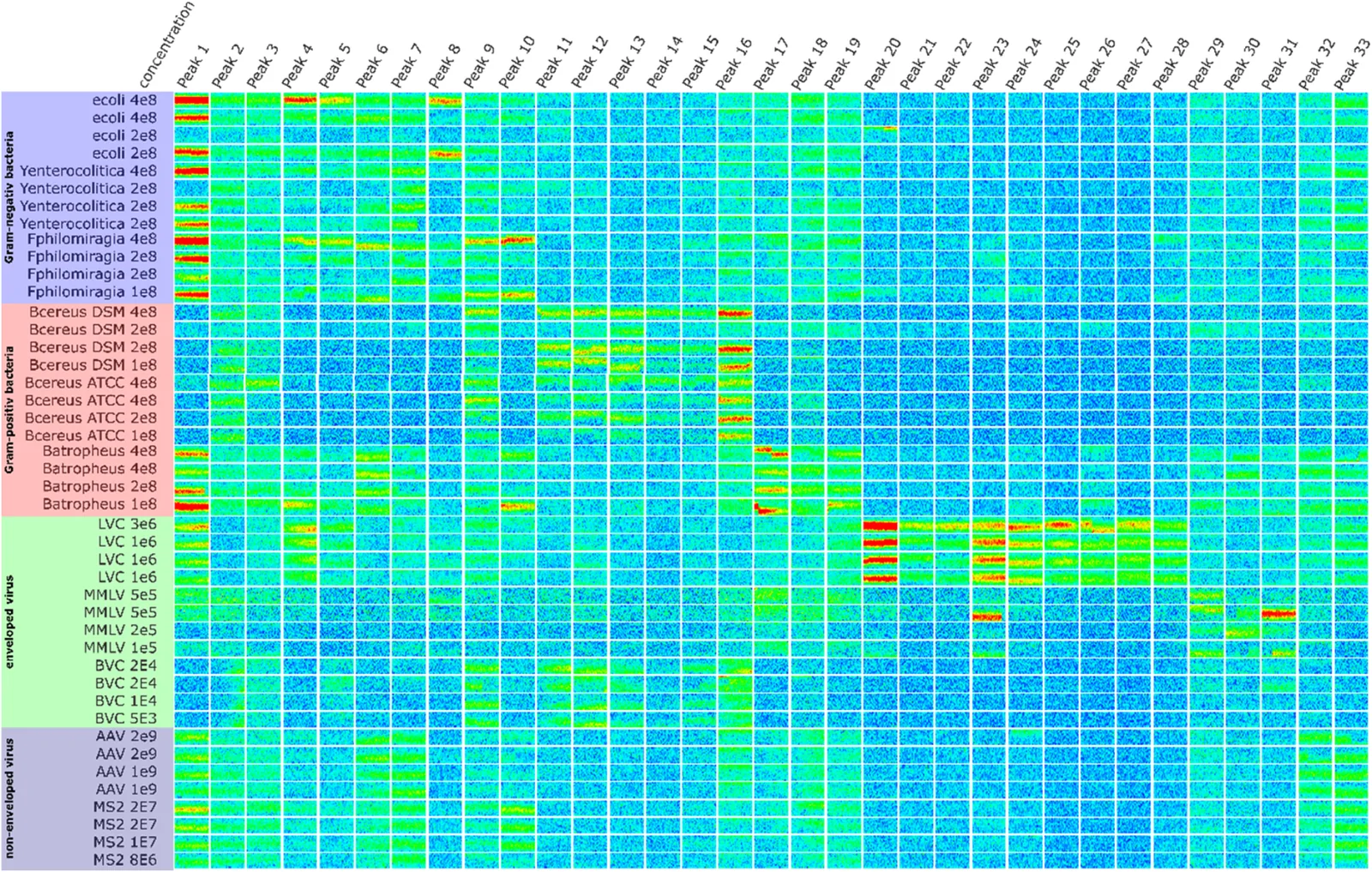
Comparison of 33 selected peaks (features) from the GC–IMS data of the 11 biological targets that show great variation in intensity in the positive polarity from target to target. The slight variations between the measurements of the same bacteria are due to differences in the sample amount picked up by the ferromagnetic wire. Each of the biological samples is present with at least four different measurements from different colonies and at different concentrations.

Furthermore, we analyzed two distinct strains of *B. cereus*. Their Py–GC–IMS 2D-chromatogram showed highly similar peaks in both positive and negative polarities, confirming that strain-level variability did not obscure their shared species-level signature. Importantly, these fingerprints were clearly distinguishable from those of the closest related species included in our panel, *B. atrophaeus*. This demonstrates that Py–GC–IMS can differentiate between closely related species while maintaining consistency across strains of the same species.

The required level of identification depends on the intended application. For rapid screening and early warning, species-level identification is often sufficient to trigger an alert and initiate appropriate response measures, whereas definitive characterization and strain discrimination can subsequently be performed using reach-back techniques such as PCR, genomic sequencing, or MALDI-TOF. Compared to existing approaches, Py–GC–IMS offers a compromise between analytical performance, response time, sample preparation, and field deployability. Lateral-flow assays are rapid but rely on target-specific antibodies, whose development is laborious and requires prior knowledge of the biological agent.^47,48^ PCR, and the faster version LAMP, can provide results in approximately 30–60 min, but require sample preparation and nucleic-acid extraction, increasing the overall analysis time.^49^ MALDI-TOF often requires bacterial culture and is not readily deployable due to its vacuum requirements.^29^ In contrast, Py–GC–IMS provides results within 16 min without prior sample extraction. The subsequent GC separation reduces matrix interferences and separates the generated pyrolysis products before IMS analysis, providing an additional dimension of selectivity and chemical information. The current GC run time of 16 min, together with the time required for cleaning and system conditioning, limits the current setup to be employed in field applications that require rapid response. However, several approaches could shorten the overall analysis time. These include using shorter, narrow-bore GC columns, faster temperature ramps, higher carrier-gas flow rates, low thermal mass for column heating, and optimized purge, backflush, and cleaning procedures. Dedicated hyper-fast GCs may reduce the chromatographic separation to only a few minutes.^50–54^ However, the current setup is just a TRL3–4 demonstrator (self-built pyrolyzer and self-built dual polarity ion mobility spectrometer) developed to show basic feasibility of detecting bacteria and viruses by Py–GC–IMS. Therefore, reducing analysis time was not the primary focus of our study.

Chemical assignment of discriminative features would provide additional insight into the origin of the observed fingerprints. However, the main objective of this work is to demonstrate basic feasibility of detecting bacterial and viral agents by Py–GC–IMS without identifying the underlaying chemical features. This would require coupling the PY–GC–IMS to MS, which is beyond the scope of this work. Nevertheless, in a recently published study^55^ in which the same bacterial species were analyzed by Py–GC–MS, several pyrolysis products were tentatively identified as fatty acid methyl esters (FAMEs), which have also been reported by other studies.^56,21,24^ On the other hand, different studies have proposed that pyrolysis fragments originating from amino acids and carbohydrates also contribute to the overall fingerprint.^57,26,27^ However, the ML/DL analysis performed in that work suggested that classification is driven by a broader set of compounds and not by FAMEs alone. Given the molecular complexity of microorganisms, the discriminatory information likely originates not only from the presence or absence of specific peaks but also from the relative intensity patterns of multiple compounds. Comprehensive peak-assignment is therefore beyond the scope of the work, but an important topic that deserves further investigation in future work.

To partially identify some peaks, some short-chained fatty acids, namely propionic acid, butyric acid, and pentanoic acid, were measured as expected pyrolysis products for bacteria.^56^ Measurements were repeated 5 times, with the vial being weighed before and after each measurement, as shown in Table S3. The 2D-chromatograms can be found in the Supporting Information under Figure S17, and the average value and standard deviation of the retention time, the average value and standard deviation of the reduced ion mobility, and the average value and standard deviation of the peak amplitude are shown in Table S4, with an average amplitude variation of 16.3%. We also analyzed the peak amplitude for each spectrum for the fatty acid mixture shown in Figure S18. Here, the amplitude of each analyte peak just shows slight variation, which corresponds to the sample loaded on the ferromagnetic wire. Meuzelaar et al. demonstrated in their work that a variation in sample load of 10–20% has no significant impact on the qualitative and quantitative composition of the chromatograms.^58^ This also applies to our experiments with 24.25% RSD for sample loading and a standard deviation for the amplitude of 16.30%. The retention time and reduced ion mobility have a standard deviation of 0.01% and 0.2%, respectively. The measurement error of the retention time is, with 0.01%, just slightly higher than the GC specifications stating 0.008%.

The identification capability of such a device relies on the quality and size of the reference database. As the number of target organisms increases, direct comparison of fingerprints becomes increasingly challenging. Besides accurate identification, rapid analysis is particularly important for BWA detection, where timely recognition of the corresponding bacterium or virus is essential. As the number of target organisms increases, direct comparison approaches may eventually reach their limits. Therefore, two unsupervised ML baselines, PCA and HCA, were conducted on a representative subset containing measurements from two bacteria (*F. philomiragia* and *B. cereus*) and two viruses (LVC and AAV).

PCA was performed using the combined positive- and negative-ion Py–GC–IMS data, resulting in clear separation of all four classes, as shown in Figure 8. The distinct clustering observed within the space defined by the first three principal components indicates that the pyrolysis products generated from each microorganism produce characteristic ion mobility patterns. These differences likely originate from variations in the biochemical composition of the organisms, including proteins, lipids, nucleic acids, and cell-envelope structures, which generate different volatile decomposition products during pyrolysis.

**Figure 8:**
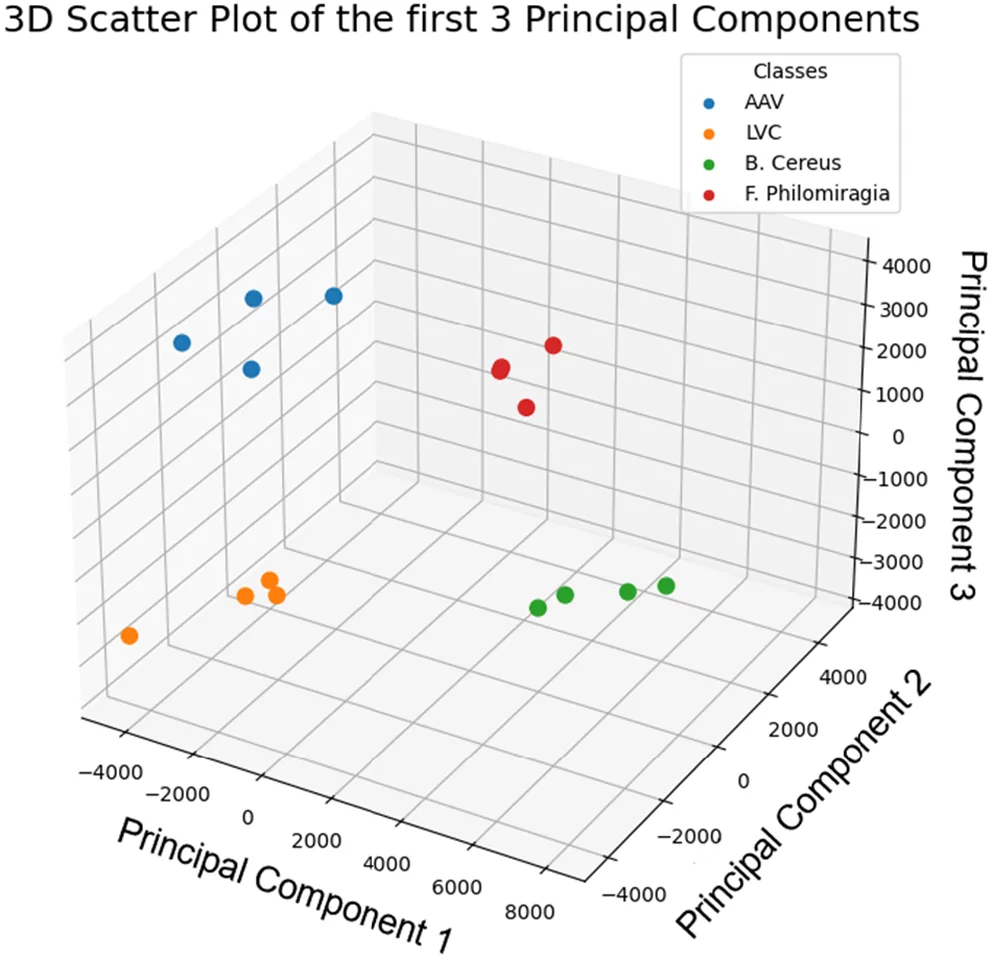
PCA 3D projection using the first three principal components.

As shown in Figure S19, the first 14 principal components explain 95% of the cumulative variance. The contribution of multiple principal components suggests that class discrimination is not governed by a single dominant marker compound but rather by a combination of chemical features distributed throughout the fingerprint profile. This observation is consistent with the complex nature of pyrolysis products, where numerous compounds collectively contribute to the analytical signature.

Figure S20a–d present the mean values and standard deviations of the first 14 principal components for each class. The observed differences indicate that the extracted features capture systematic variations in the underlying chemical fingerprints of the bacteria and viruses. The use of both ion polarities further increases the available chemical information by detecting compounds that preferentially form either positive or negative ions, thereby improving class discrimination.

Subsequently, HCA was applied to evaluate similarities among the measurements. As illustrated in Figure S21, samples belonging to the same organism form distinct clusters, while measurements acquired at identical concentration levels are frequently grouped together. This behavior indicates that the fingerprints are reproducible and that the clustering reflects genuine chemical similarities rather than random measurement variation.

Although PCA and HCA are unsupervised methods, the results demonstrate that the Py–GC–IMS fingerprints contain sufficient chemically relevant information to distinguish between different microorganisms. The observed clustering is therefore not merely a statistical effect but also reflects differences in the composition of the pyrolysis products generated from each organism. These findings are consistent with the visual differences observed in the peak distribution fingerprints presented in Figure 7 and further support the key conclusions of this work. Future work will focus on identifying the specific features that contribute most strongly to class separation and on evaluating supervised machine-learning approaches for classification in larger and more diverse databases.

## Conclusion

In this work, we use a self-built pyrolysis based on the Curie point method coupled to a GC–IMS with an ultra-fast polarity switching IMS to identify different bacteria and viruses. The IMS allows for simultaneous recording of both ion polarities in one single GC run. With the measured bacteria, 2 Gram-positive bacteria, including 2 strains of the same bacteria, and 3 Gram-negative bacteria, as well as viruses, 3 enveloped and 2 nonenveloped viruses, a good representation of different types of bacteria and viruses has been analyzed. By separating the volatile constituents of the bacteria and viruses in two dimensions (GC retention time and IMS drift time), a clear distinction of all tested bacteria and viruses can be achieved. When comparing the peaks (features) one by one, differences exist, including distinct peaks that exclusively represent one species. The examples shown here demonstrate the promising potential of Py–GC–IMS for identifying biological agents in a reasonable time frame and with reasonable effort. For the future, the GC run time can be shortened by either shortening the column length while using narrow-bore columns and increasing the heating ramp of the GC. Furthermore, dedicated Hyper-fast GC might be an option. Comparing the selected features giving the fingerprints, there are at least a couple of differences between all of the measured biological samples, while repeated measurements of the same biological sample from different colonies at different concentrations show identical fingerprints. Further, unsupervised ML-algorithms were used, showing promising results.

Thus, the presented data of Py–GC–IMS render a promising approach for fast detection of biological agents. Future work will focus on in-depth ML and DL analysis, which could reveal confident identification of targets at lower concentrations and in a much larger data set, using a more diverse set of classes and a larger number of experiments per class.

## Supplementary Material



## Notes

Sectional View of the pyrolyzer (Figure S1); used bacteria and viruses with their origins (Table S1); weight differences of the glass vials before and after measurements (Table S2); 2D-chromatogram of a clean ferromagnetic sample holder (Figure S2); 2D-chromatograms of tap-, DI- and lipid-free ultra-pure water (Figures S3–5); 2D-chromatograms of Gram-negative and Gram-positive bacteria (Figures S6–11); 2D-chromatograms of enveloped and nonenveloped viruses (Figures S12–S15); comparison of the selected negative peaks (Figure S16); weight before and after dipping the ferromagnetic vial into the fatty acids (Table S3); 2D-chromatogram of the fatty acids (Figure S17); 2D-chromatogram with the maximum amplitudes of the fatty acids (Figure S18); average retention time, drift time, and amplitudes of the fatty acids (Table S4); part of the principal component analysis (Figure S19); average and standard deviation of the principal component analysis (Figure S20); hierarchical clustering dendrogram (Figure S21)­(PDF)
