# Supplementary material for: Identification of Bacteria and Viruses by Pyrolysis–Gas Chromatography–Ion Mobility Spectrometry
Source: Anal Chem. 2026 Sep 2;98(36):26998–7008. doi: 10.1021/acs.analchem.6c04057 (PMC13584632; doi:10.1021/acs.analchem.6c04057)
Supplement: Supplementary file 1 [file ac6c04057_si_001.pdf]

**Supporting Information**

# Identification of Bacteria and Viruses by Pyrolysis-Gas Chromatography-Ion Mobility Spectrometry

Daniel Röckrath[1,*], Moritz Hitzemann[1], Alejandra Vargas Valderrama[2,3], Arne Höhne[1], Maryam Damen[1], Georgios Kirtsanis[4], Georgios Dolias[4], Martin Lippmann[1], Ayke Eckhardt[1], Christian Thoben[1], Guillaume Lepage[3], Stefan Zimmermann[1]

[1]Leibniz University Hannover, Institute of Electrical Engineering and Measurement Technology, Appelstr. 9A, 30167 Hannover, Germany

[2]Belgian Defence Laboratories (DLD), Quartier Major Housiau, Martelarenstraat 181, 1180 Vilvoorde, Belgium

[3]Royal Military Academy (RMA), Avenue de la Renaissance 30, 1000 Brussels, Belgium

[4]Information Technologies Institute (ITI), Centre for Research and Technology Helias (CERTH), 6th Km Charilaou - Thermis Rd, 57001 Thermi, Thessaloniki, Greece

*Corresponding author

Daniel Röckrath, E-mail: daniel.roeckrath@geml.uni-hannover.de

Table of Contents

Figure S1: Sectional view of the pyrolyzer with the inner adapter (a) and the main part (b). ................3
Table S1: Bacteria, viruses, and their origins used in the study. For measurement, the bacteria were diluted mostly to a concentration of 4e8 cfu/µL. While the viruses stayed at their original concentration, due to them being mostly at quite low concentrations. All biological agents were diluted by half and by a quarter afterward for further measurements. ................................................4
Table S2: Start and end weight of the glass vial containing a sample mixture of water and bio sample, and the difference in µg that sticks to the ferromagnetic wire.........................................................4
Figure S2: 2D-chromatogram of a clean ferromagnetic sample holder measured with the Py-GC-IMS. 5
Figure S3: 2D-chromatogram of tap water measured with the Py-GC-IMS ..........................................5
Figure S4: 2D-chromatogram of DI-water measured with the Py-GC-IMS............................................6
Figure S5: 2D-chromatogram of lipid-free ultra-pure water measured with the Py-GC-IMS.................6
Figure S6: 2D-chromatogram of the Gram-negative bacterium Escherichia coli measured with the PY-GC-IMS at a concentration of 2e8 cfu/µl (6e7 cfu)...........................................................................7
Figure S7: 2D-chromatogram of the Gram-negative bacterium Yersinia enterocolitica at a concentration of 2e8 cfu/µl (6e7 cfu). ..............................................................................................7
Figure S8: 2D-chromatogram of the Gram-negative bacteria Francisella philomiragia at a concentration of 4e8 cfu/µl (1e8 cfu). ..............................................................................................8
Figure S9: 2D-chromatogram of the Gram-positive bacteria Bacillus cereus (DSM002302) at a concentration of 4e8 cfu/µl (1e8 cfu). ..............................................................................................8
Figure S10: 2D-chromatogram of the Gram-positive bacteria Bacillus cereus (ATCC14579) at a concentration of 4e8 cfu/µl (1e8 cfu). ..............................................................................................9
Figure S11: 2D-chromatogram of the Gram-positive bacteria Bacillus atropheus at a concentration of 4e8 cfu/µl (1e8 cfu). ...........................................................................................................................9
Figure S12: 2D-chromatogram of the enveloped virus Murine leukemia virus at a concentration of 4.68e5 tu/µl (1e5 tu). ........................................................................................................................10

Figure S13: 2D-chromatogram of the enveloped virus Baculoviridae at a concentration of 2.27e4 pfu/µl (7e3 pfu). ....................10
Figure S14: 2D-chromatogram of the non-enveloped virus Adeno-associated viruses at a concentration of 2e9 gc/µl (8e8 gc). ....................11
Figure S15: 2D-chromatogram of the non-enveloped virus Bacteriophage MS2 at a concentration of 1e7 pfu/µl (6e6 pfu). ....................11
Figure S16: Comparison of the 11 selected negative peaks (features) with the greatest variation in intensity. ....................12
Table S3: The weight before and after dipping the ferromagnetic vial into the fatty acids mixture of propionic acid, butyric acid and pentanoic acid with an average difference of 502 µg, a standard deviation of 109 µg and a resulting RSD of 21.74 %. ....................13
Figure S17: 2D-chromatogram of the fatty acid mixture consisting of propionic acid, butyric acid, and pentanoic acid. ....................13
Figure S18: Maximum amplitude of the 2D-chromatogram excluding the reactant ion peak over the retention time with 5 repeated measurements of fatty acids: propionic acid, butyric acid, and pentanoic acid. ....................14
Table S4: The average retention time, drift time, and amplitude with their respective standard deviation of the measured fatty acids. ....................15
Figure S19: Cumulative explained variance ratio by principal component analysis. ....................15
Figure S20: Average and standard deviation of each principal component for each representative class ((a): B. cereus, (b): F. philomiragia, (c): LVC, (d): AAV). ....................16
Figure S21: Hierarchical clustering dendrogram of the representative dataset. ....................17

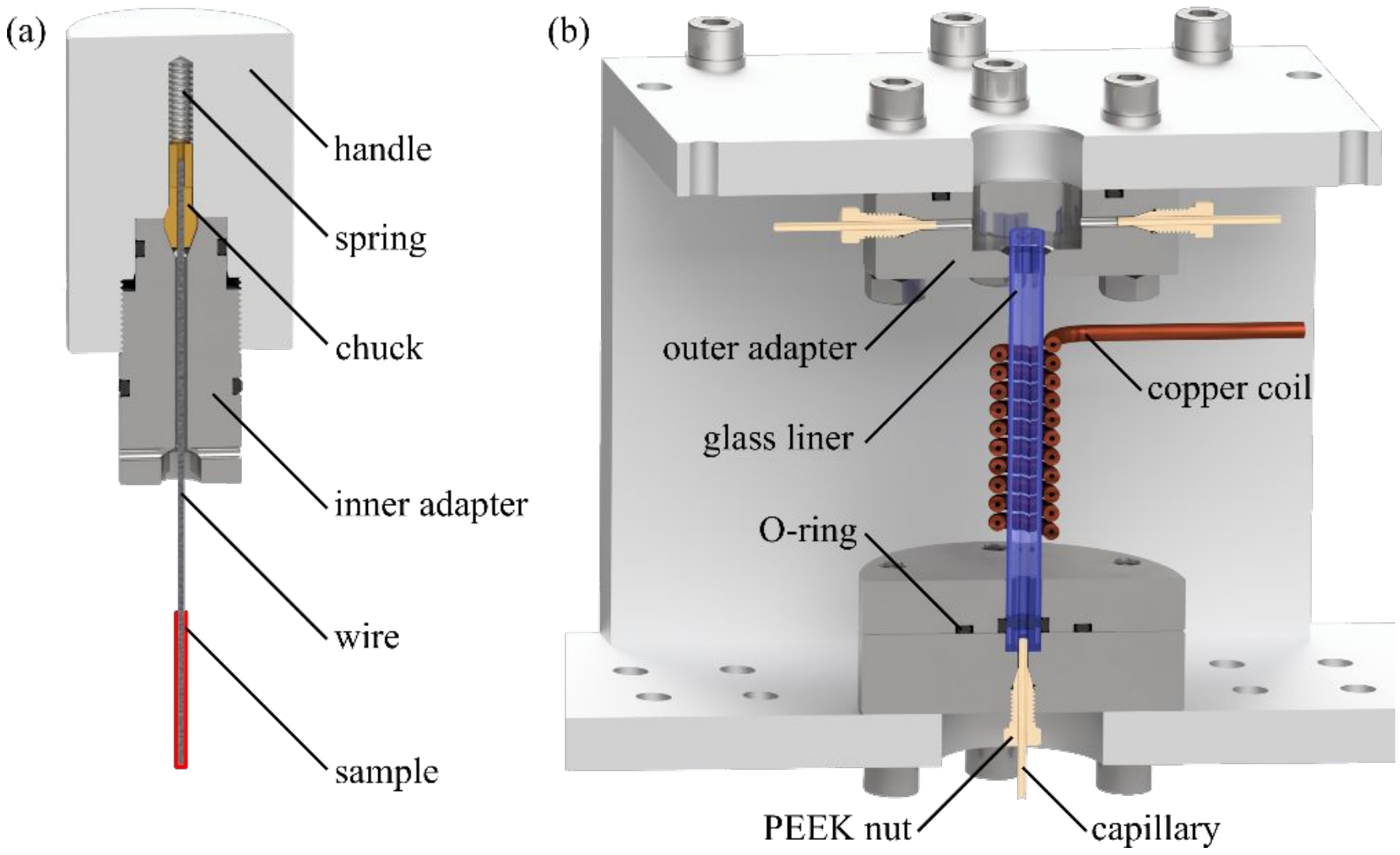


*Figure S1: Sectional view of the pyrolyzer with the inner adapter (a) and the main part (b).*

*Table S1: Bacteria, viruses, and their origins used in the study. For measurement, the bacteria were diluted mostly to a concentration of 4e8 cfu/µL. While the viruses stayed at their original concentration, due to them being mostly at quite low concentrations. All biological agents were diluted by half and by a quarter afterward for further measurements.*

| Group | Name | Other names | Concentrations | Source | Figure | Measured concentra-tions |
|---|---|---|---|---|---|---|
| Gram-negative | *Escherichia coli* | DSM500 | 1,8e11 cfu/µL | German Collection of Microorganisms and Cell Cultures GmbH, Braunschweig, Germany | 5, S3 | 4e8 cfu/µL |
| | *Yersinia enterocolitica subsp. enterocolitica* | DSM4780 | 1,8e12 cfu/µL | German Collection of Microorganisms and Cell Cultures GmbH, Braunschweig, Germany | S4 | 4e8 cfu/µL |
| | *Francisella philomiragia subsp. philomiragia* | DSM 7535 | 5,2e11 cfu/µL | German Collection of Microorganisms and Cell Cultures GmbH, Braunschweig, Germany | S5 | 4e8 cfu/µL |
| Gram-positive | *Bacillus cereus* | DSM2302 | 1,9e10 cfu/µL | German Collection of Microorganisms and Cell Cultures | 6, S6 | 4e8 cfu/µL |

| | | | | | | |
|---|---|---|---|---|---|---|
| | | | | GmbH, Braunschweig, Germany | | |
| | *Bacillus cereus* | ATCC14579 | 3,2e10 cfu/µL | ATCC, USA | S7 | 4e8 cfu/µL |
| | *Bacillus atrophaeus* | DSM675 | 5,0e11 cfu/µL | German Collection of Microorganisms and Cell Cultures GmbH, Braunschweig, Germany | S8 | 4e8 cfu/µL |
| Enveloped | Lentivirus | VB010000-9492agg | 2.61e6 tu/µl | VectorBuilder Inc. USA | 7 | 2.61e6 tu/µl |
| | Murine Leukemia viruses | VB010000-9307 ddn | 4,68e5 tu/µl | VectorBuilder Inc. USA | S9 | 4,68e5 tu/µl |
| | Baculovirus | VB010000-9305vtu | 2.27e4 pfu/ul | VectorBuilder Inc. USA | S10 | 2.27e4 pfu/ul |
| Non-enveloped | Adeno-associated virus | VB010000-9278ffw | 2.01e9 gc/µl | VectorBuilder Inc. USA | S11 | 2.01e9 gc/µl |
| | Bacteriophage MS2 | DLD-Bio | 2e7 pfu/µl | RMA-DLD | S12 | 2e7 pfu/µl |

tu: transducing units, gc: genome copies, pfu: plaque forming units

*Table S2: Start and end weight of the glass vial containing a sample mixture of water and bio sample, and the difference in µg that sticks to the ferromagnetic wire.*

| Measurement | Start weight in g | End weight in g | Difference in µg | cfu on wire with a concentration of 4e8 cfu/µl |
|---|---|---|---|---|
| 1 | 2.82524 | 2.82487 | 370 | 148e6 cfu |
| 2 | 2.81417 | 2.81387 | 300 | 120e6 cfu |
| 3 | 2.86862 | 2.86831 | 310 | 124e6 cfu |
| 4 | 2.79336 | 2.79311 | 250 | 100e6 cfu |
| 5 | 2.81524 | 2.81494 | 300 | 120e6 cfu |
| 6 | 2.86657 | 2.86635 | 220 | 88e6 cfu |
| 7 | 2.81549 | 2.81509 | 400 | 160e6 cfu |
| 8 | 2.78033 | 2.78014 | 190 | 760e6 cfu |
| 9 | 2.88068 | 2.88029 | 390 | 156e8 cfu |

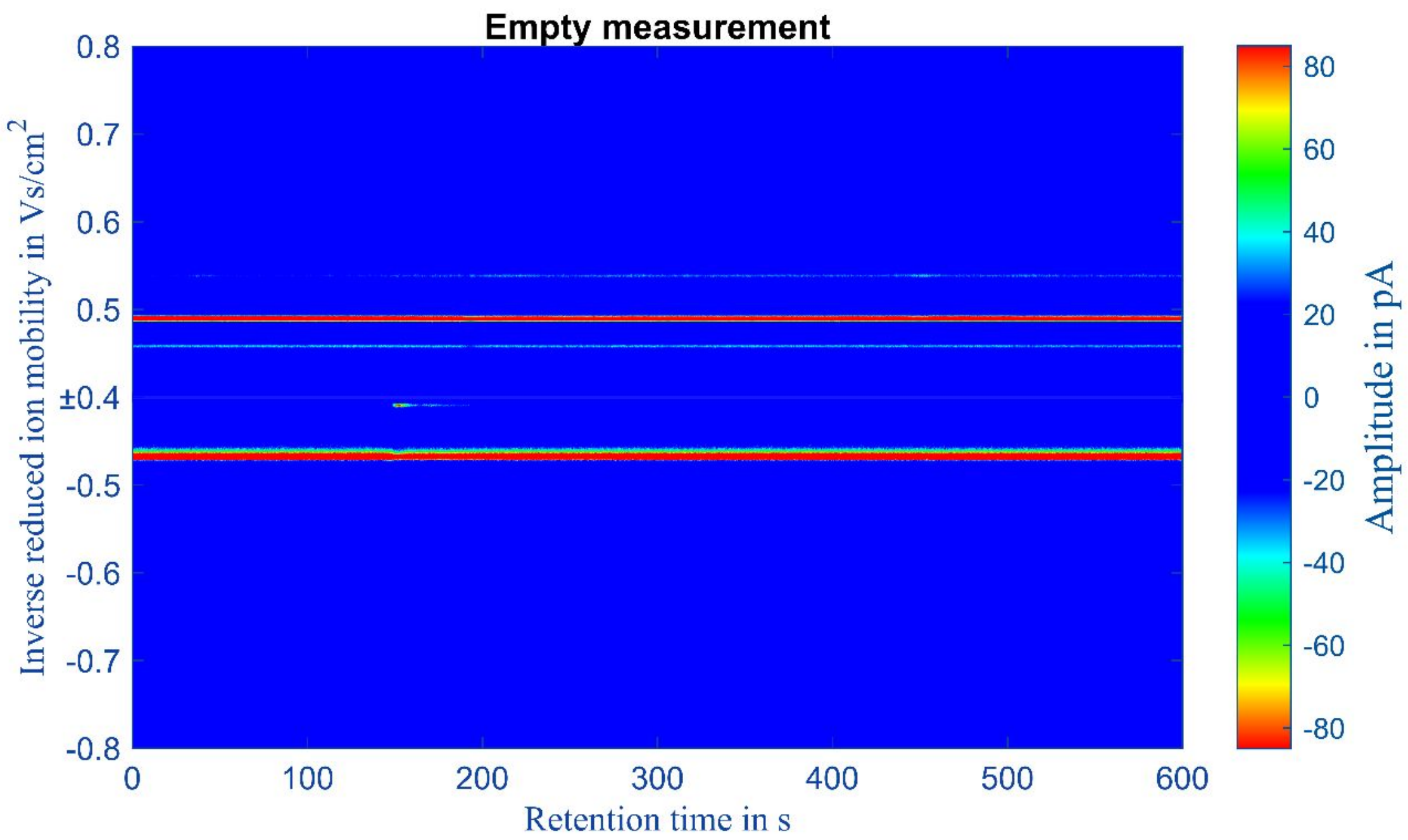


*Figure S2: 2D-chromatogram of a clean ferromagnetic sample holder measured with the Py-GC-IMS.*

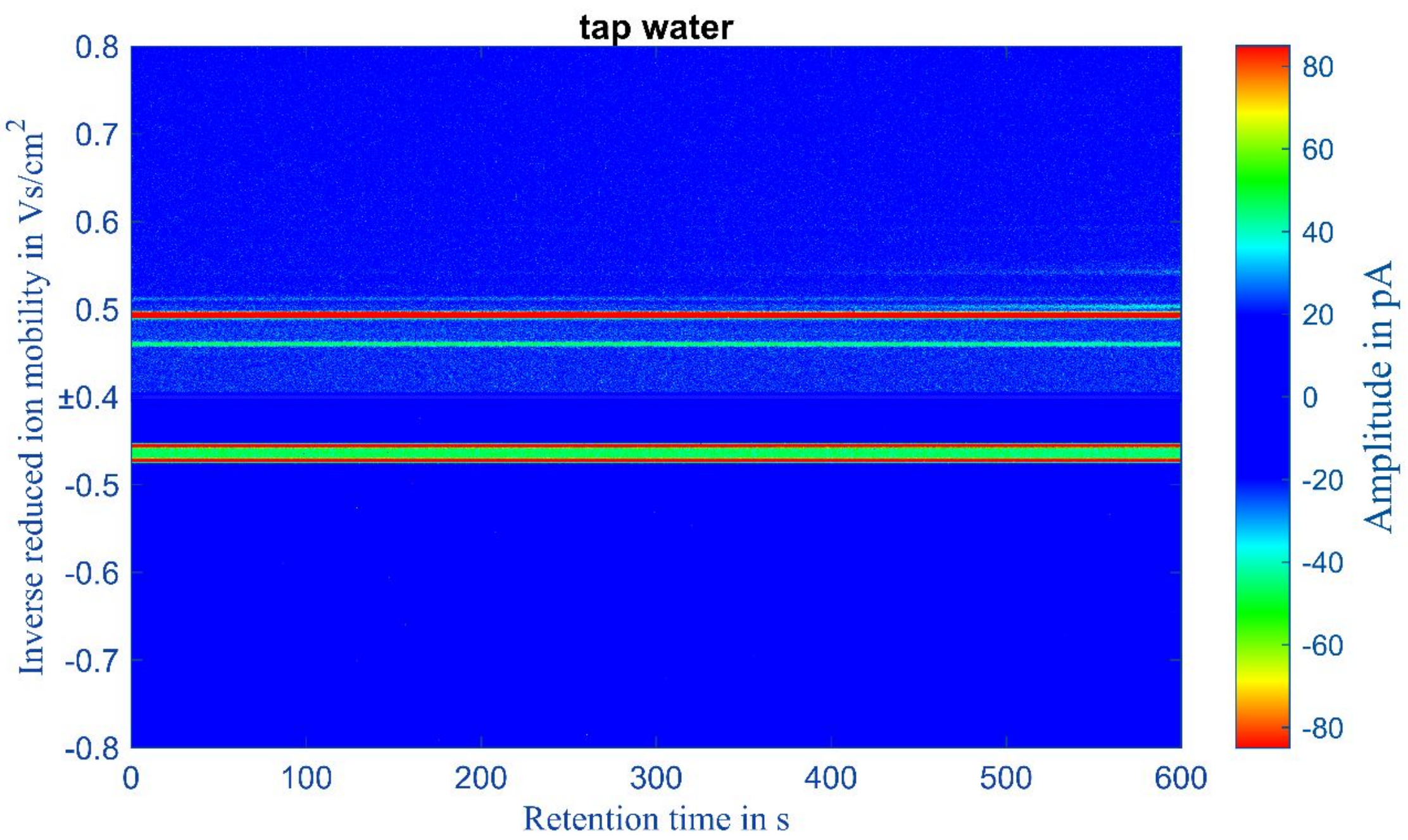


*Figure S3: 2D-chromatogram of tap water measured with the Py-GC-IMS*

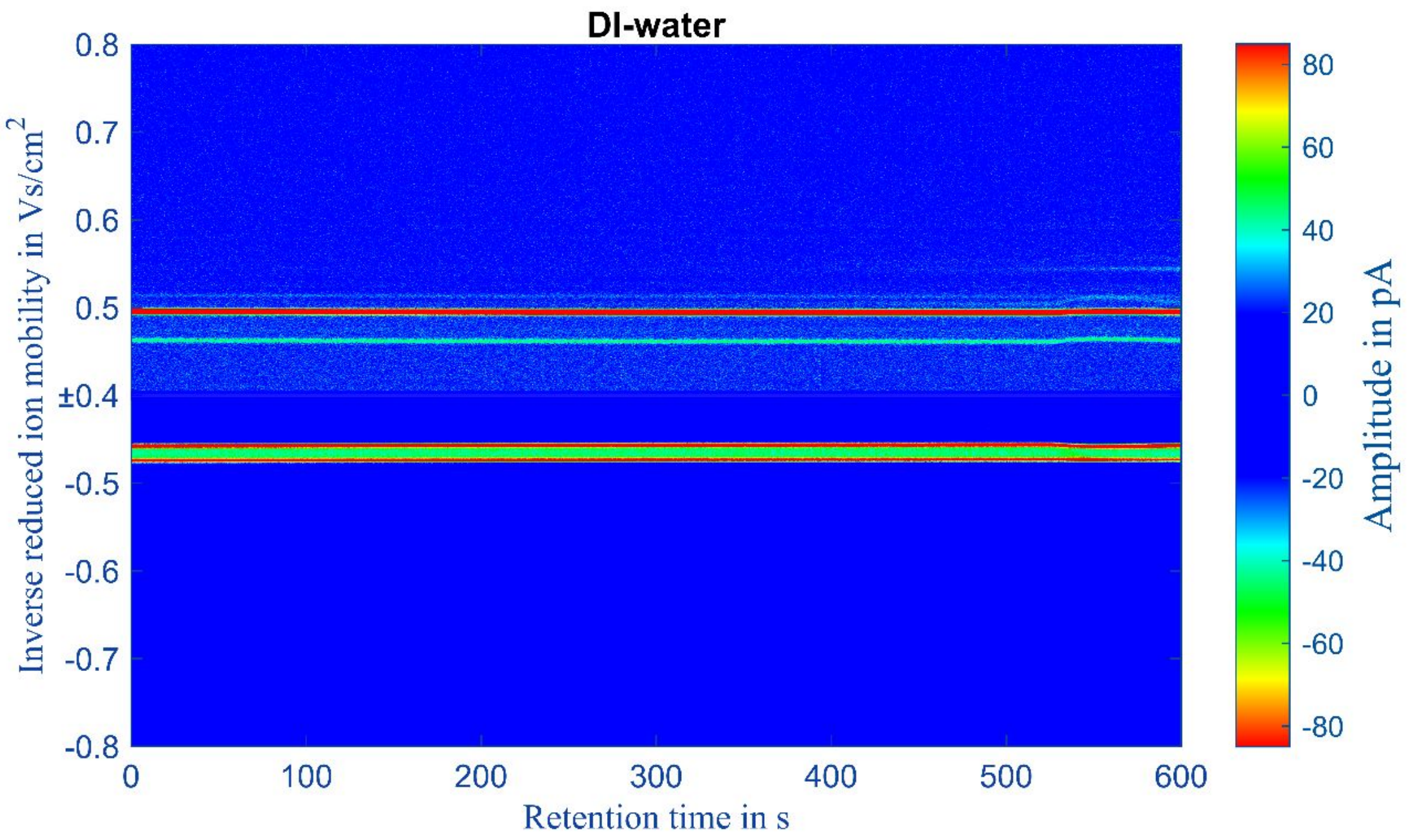


*Figure S4: 2D-chromatogram of DI-water measured with the Py-GC-IMS*

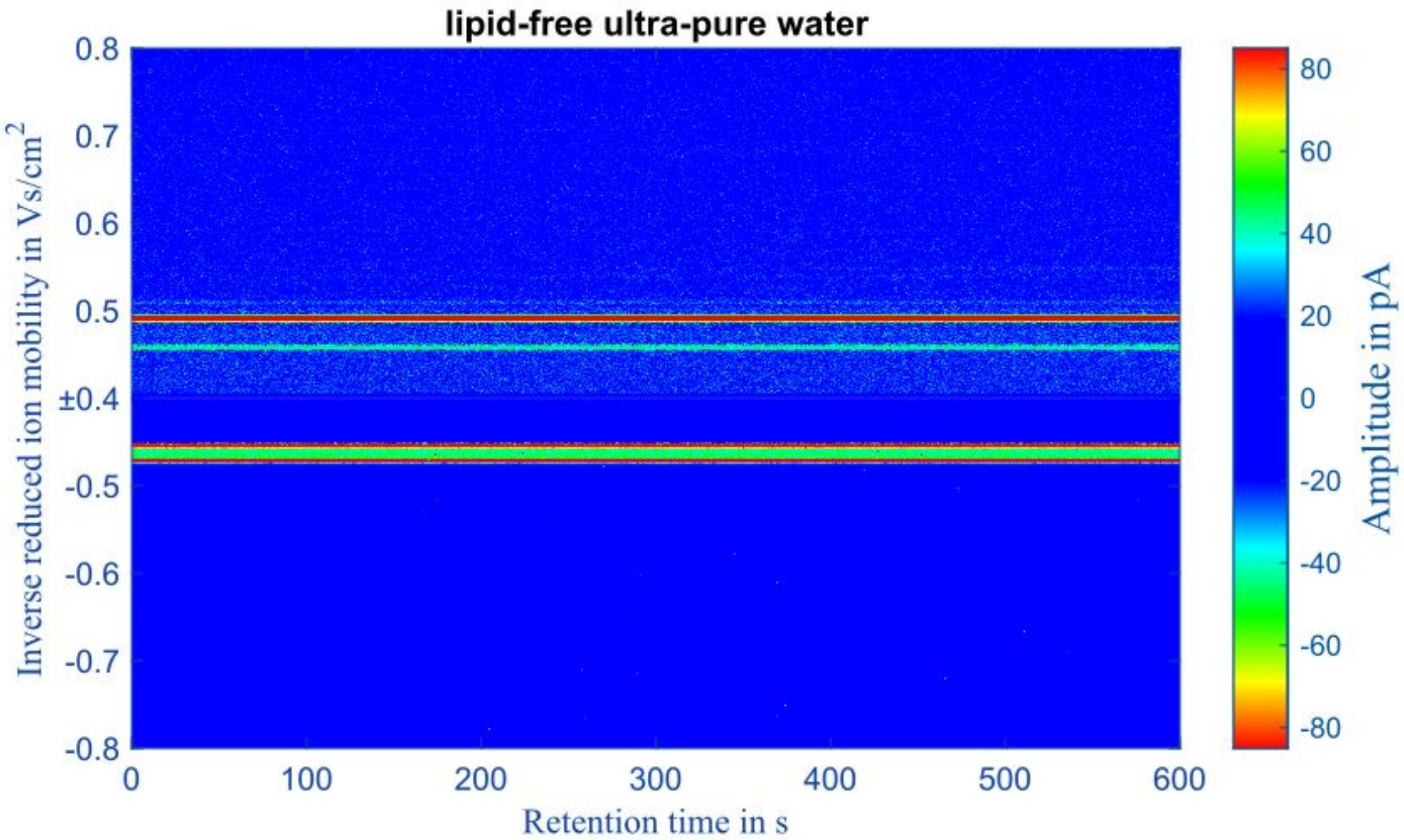


*Figure S5: 2D-chromatogram of lipid-free ultra-pure water measured with the Py-GC-IMS*

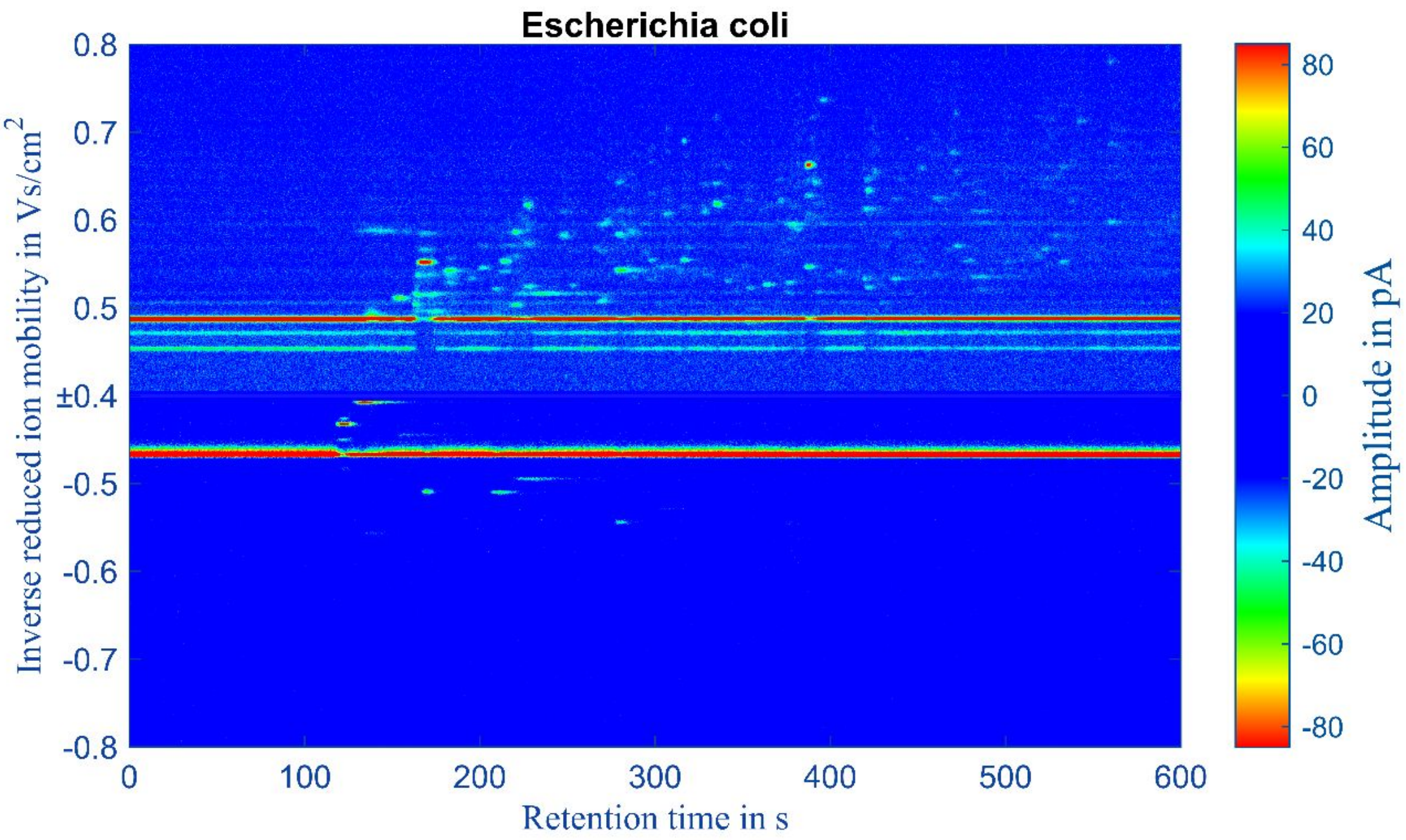


*Figure S6: 2D-chromatogram of the Gram-negative bacterium Escherichia coli measured with the PY-GC-IMS at a concentration of 2e8 cfu/µl (6e7 cfu).*

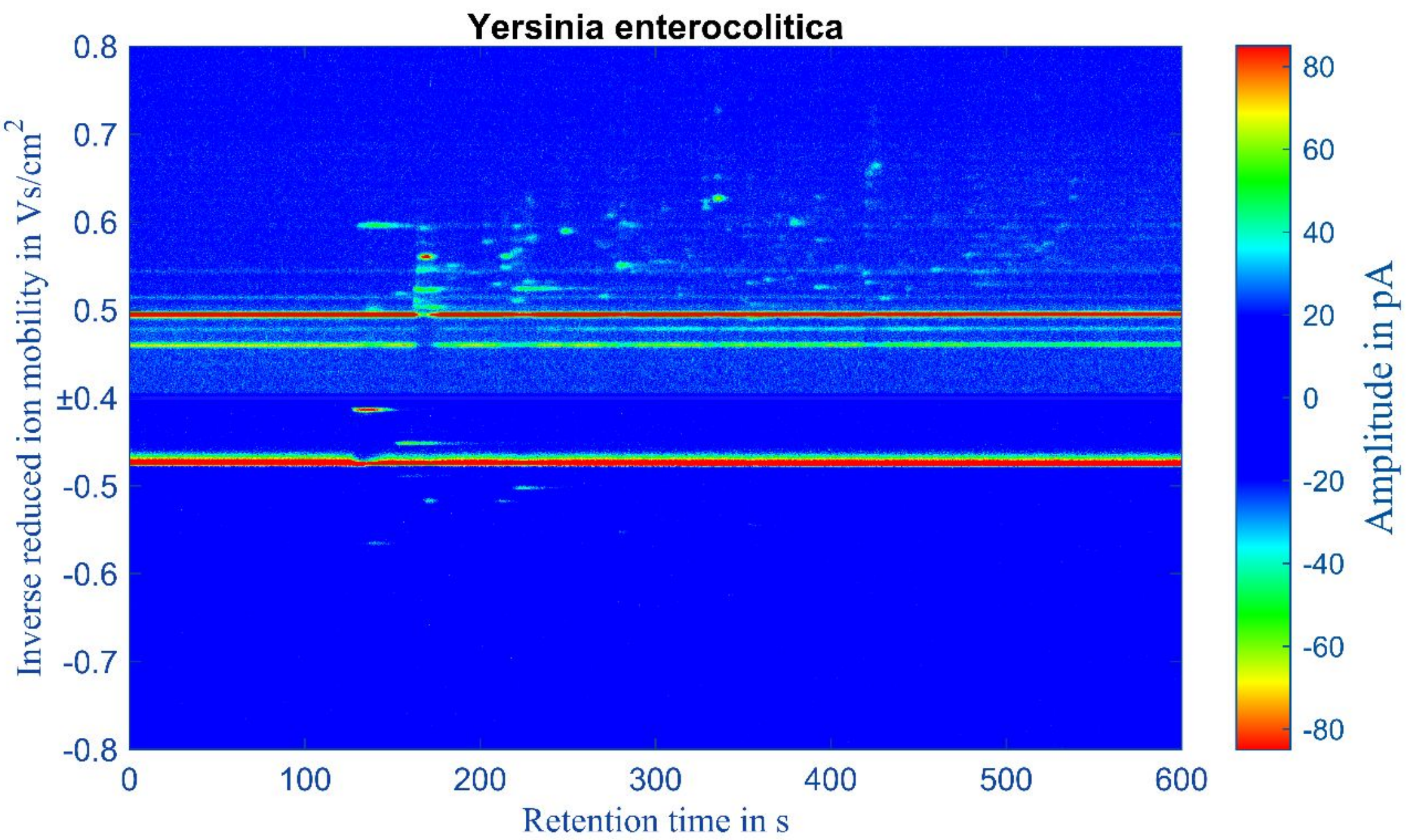


*Figure S7: 2D-chromatogram of the Gram-negative bacterium Yersinia enterocolitica at a concentration of 2e8 cfu/µl (6e7 cfu).*

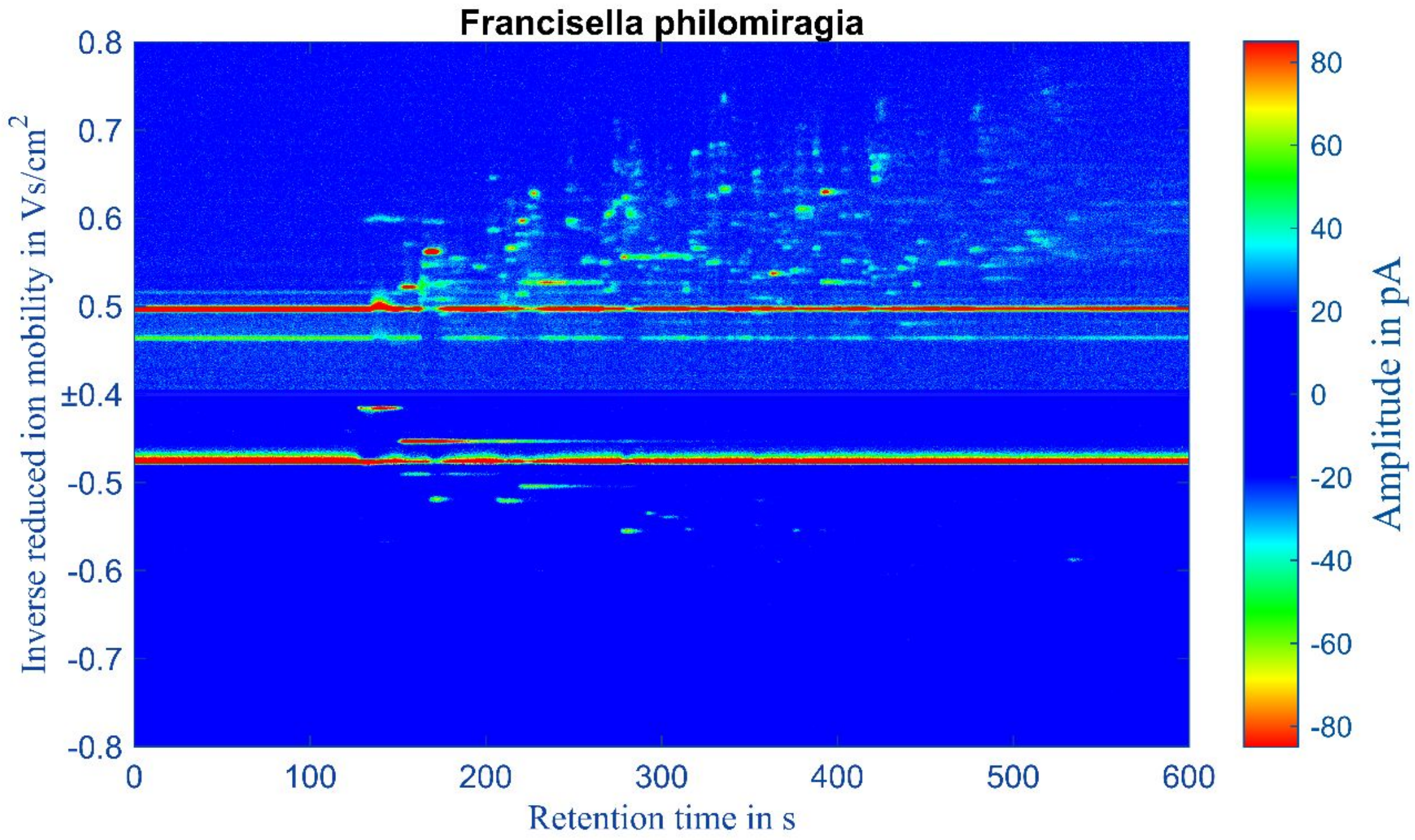


*Figure S8: 2D-chromatogram of the Gram-negative bacteria Francisella philomiragia at a concentration of 4e8 cfu/µl (1e8 cfu).*

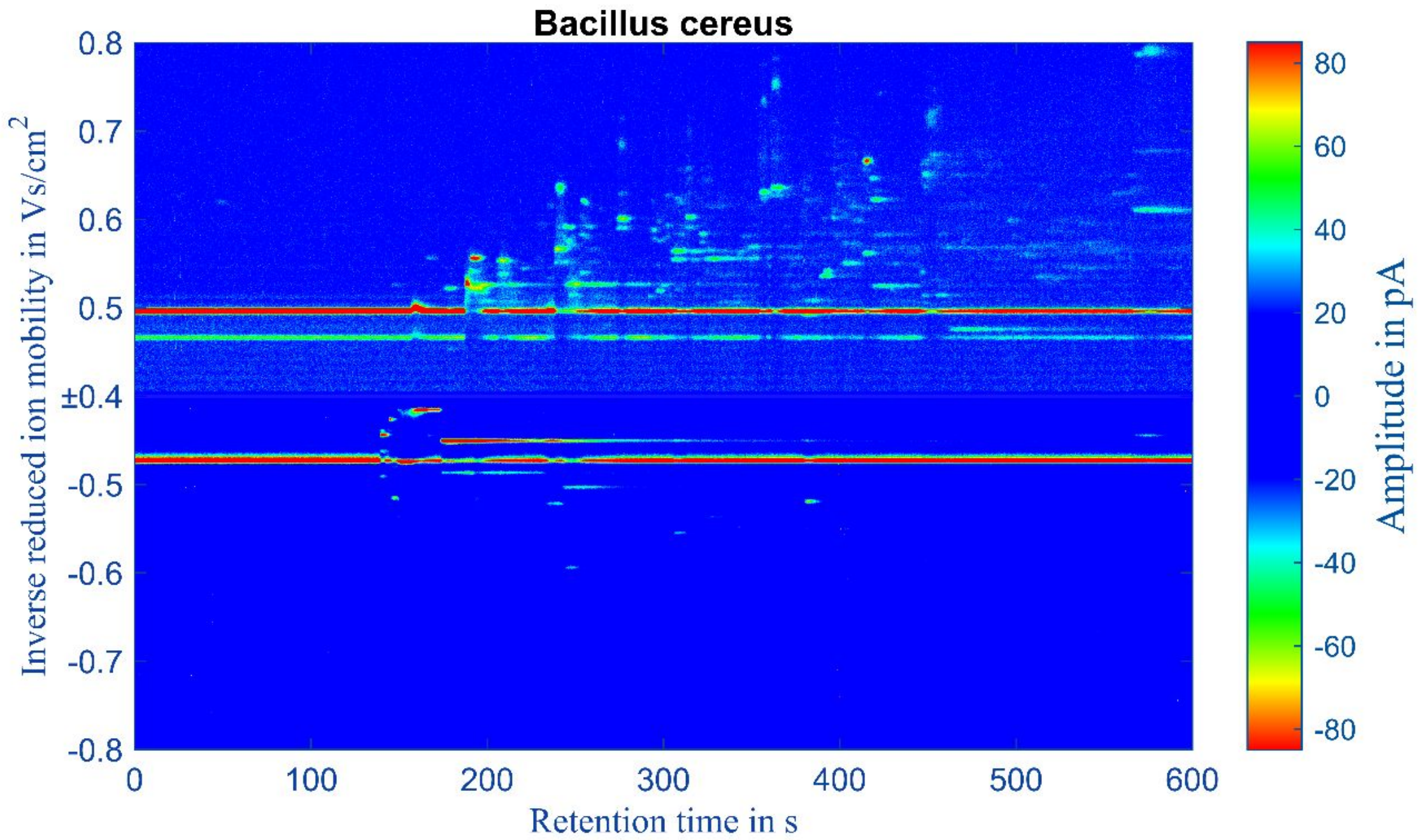


*Figure S9: 2D-chromatogram of the Gram-positive bacteria Bacillus cereus (DSM002302) at a concentration of 4e8 cfu/µl (1e8 cfu).*

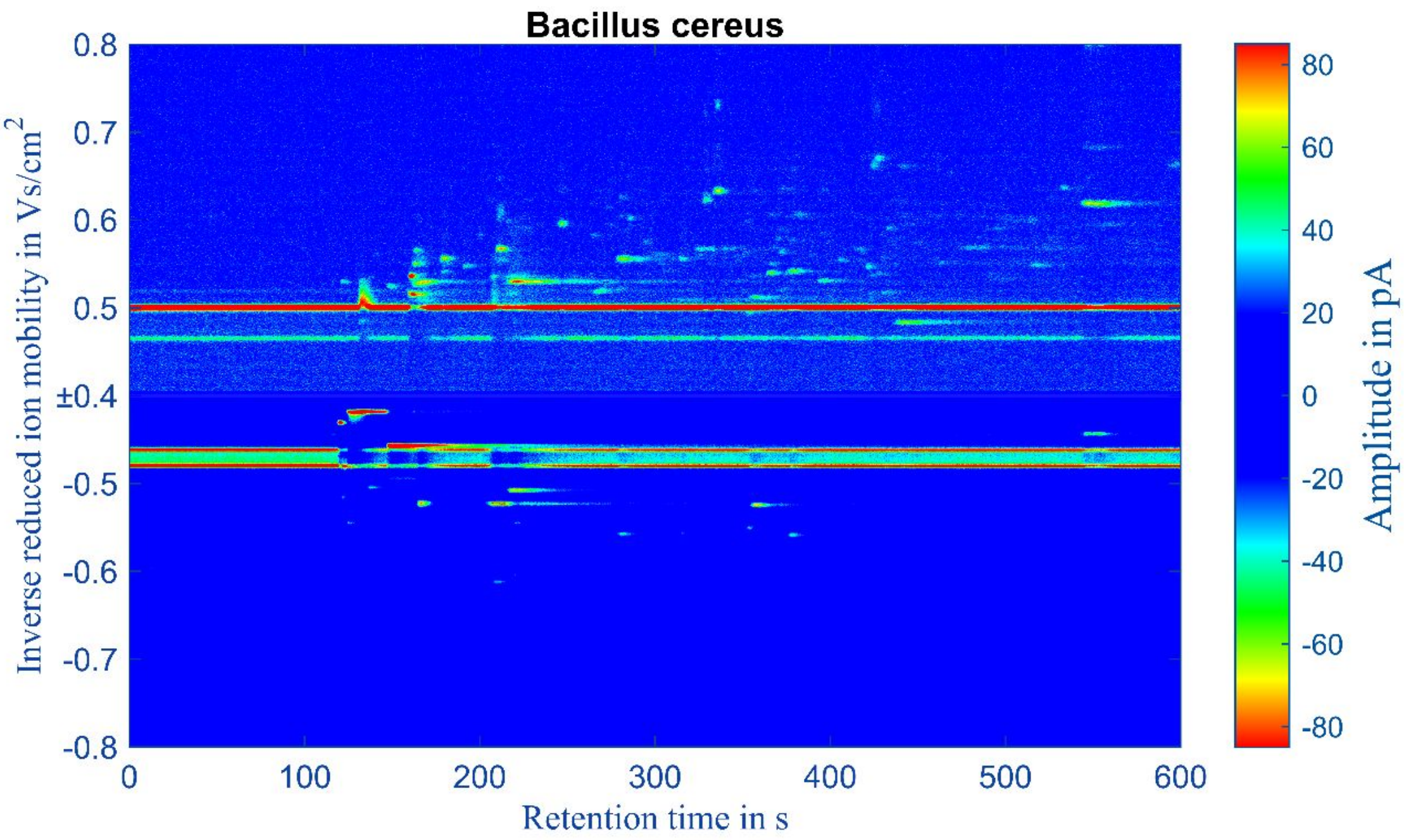


*Figure S10: 2D-chromatogram of the Gram-positive bacteria Bacillus cereus (ATCC14579) at a concentration of 4e8 cfu/µl (1e8 cfu).*

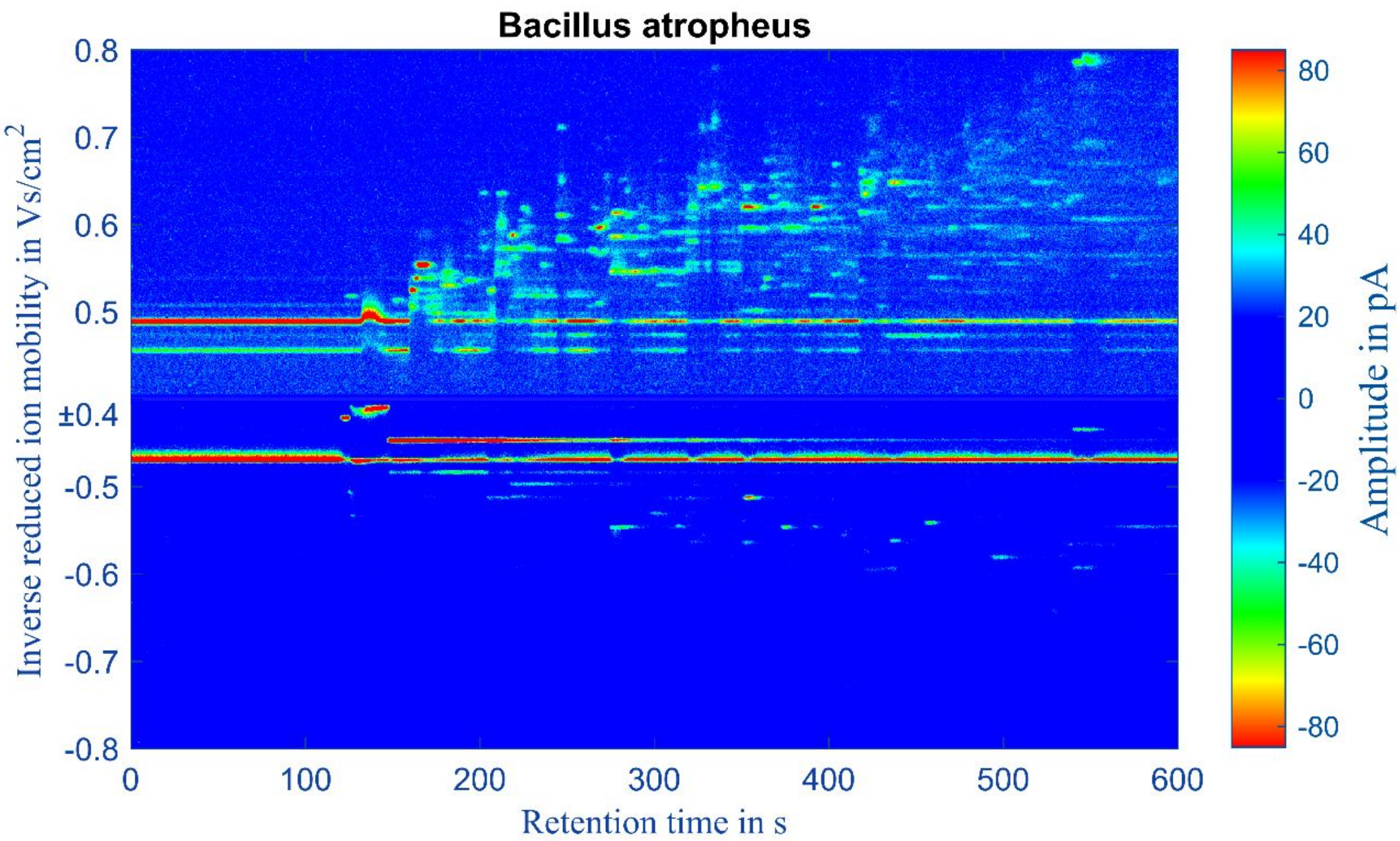


*Figure S11: 2D-chromatogram of the Gram-positive bacteria Bacillus atropheus at a concentration of 4e8 cfu/µl (1e8 cfu).*

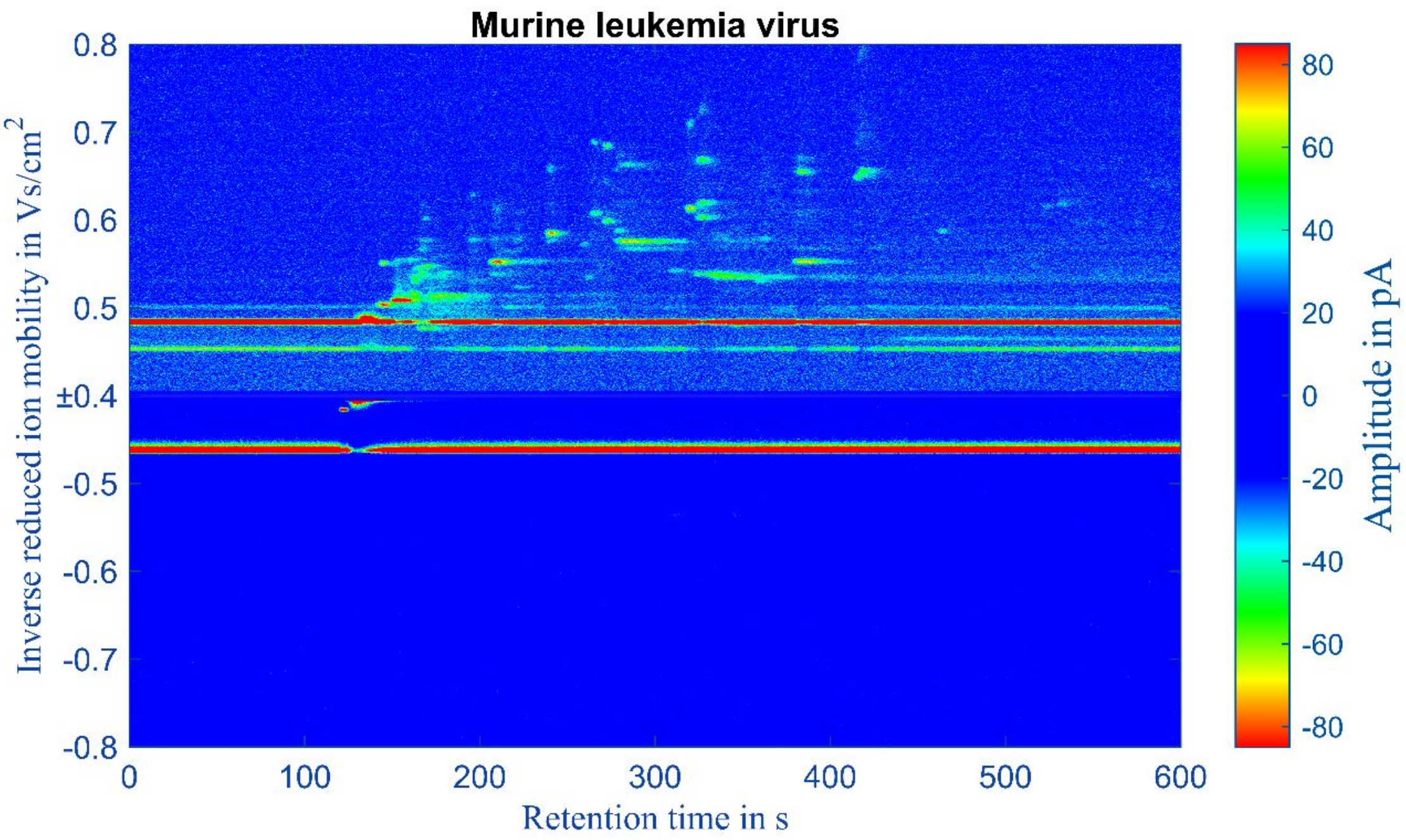


*Figure S12: 2D-chromatogram of the enveloped virus Murine leukemia virus at a concentration of 4.68e5 tu/µl (1e5 tu).*

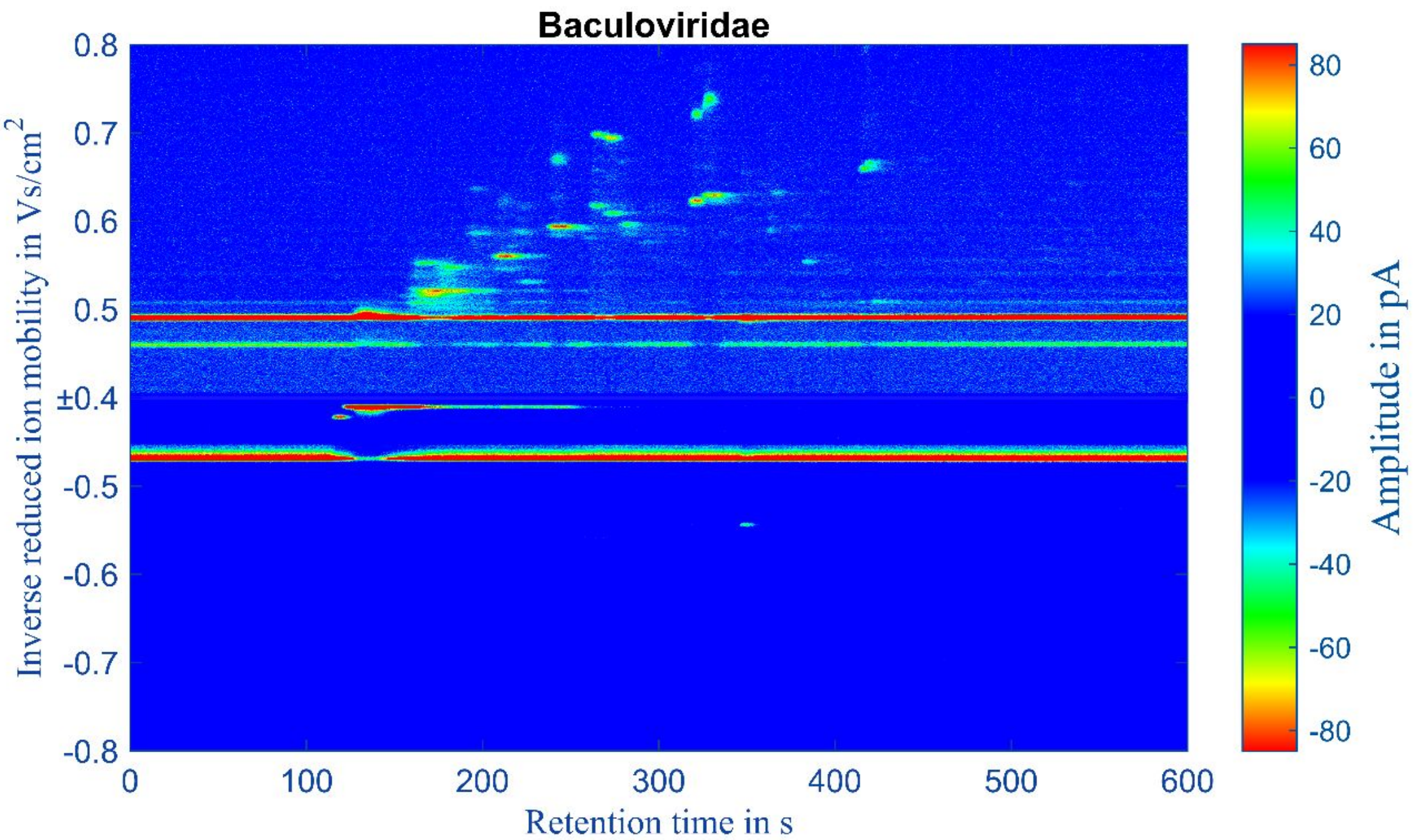


*Figure S13: 2D-chromatogram of the enveloped virus Baculoviridae at a concentration of 2.27e4 pfu/µl (7e3 pfu).*

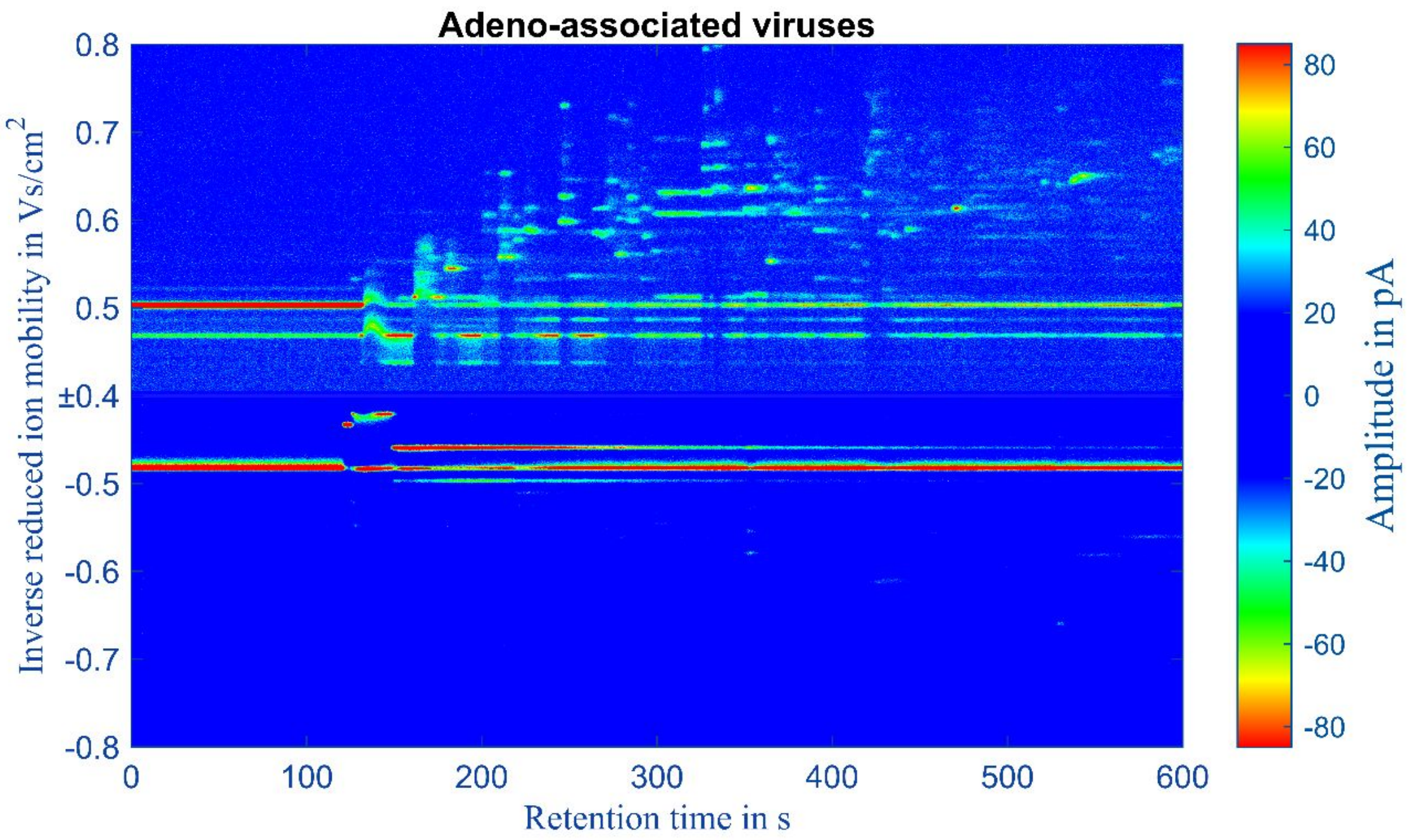


*Figure S14: 2D-chromatogram of the non-enveloped virus Adeno-associated viruses at a concentration of 2e9 gc/µl (8e8 gc).*

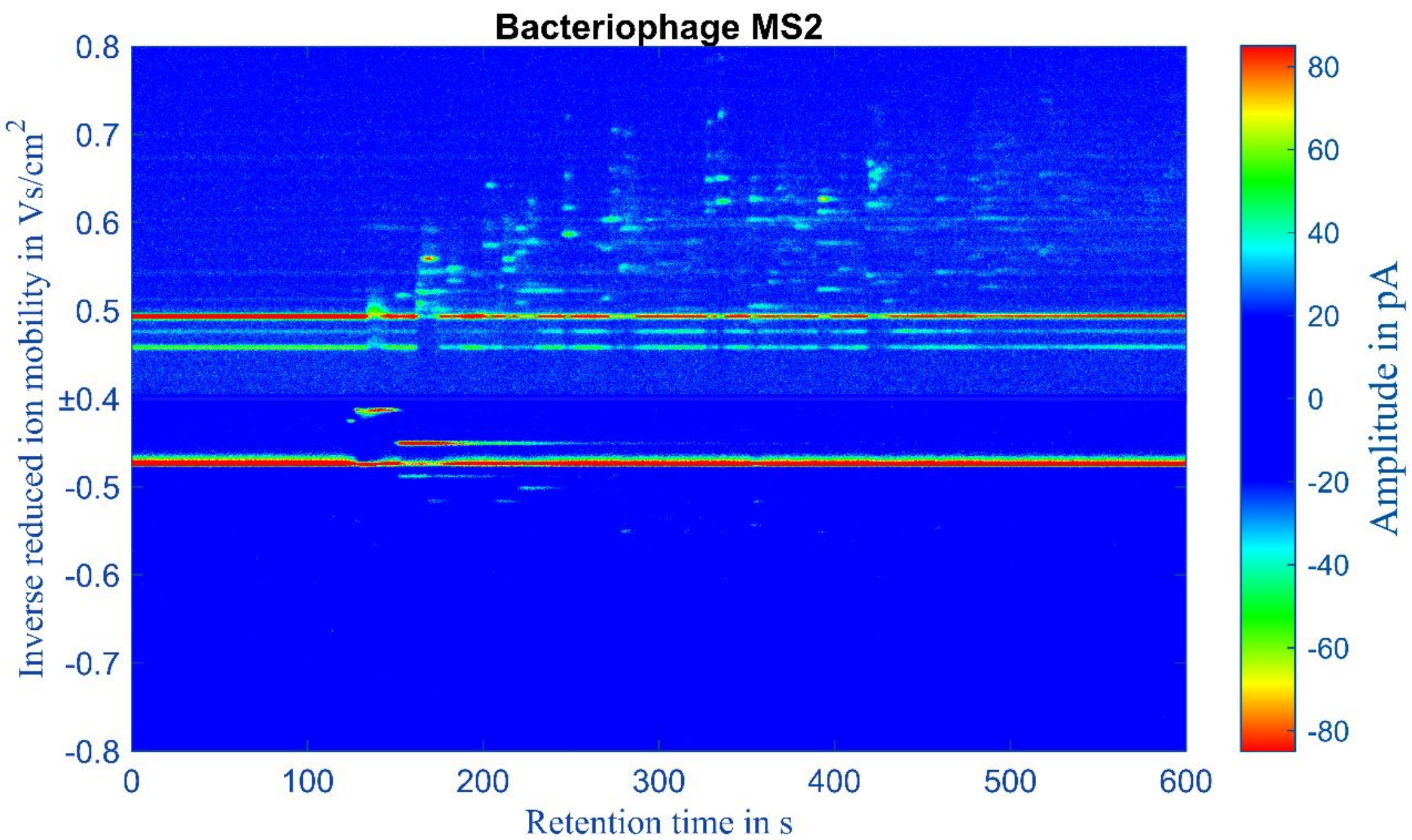


*Figure S15: 2D-chromatogram of the non-enveloped virus Bacteriophage MS2 at a concentration of 1e7 pfu/µl (6e6 pfu).*

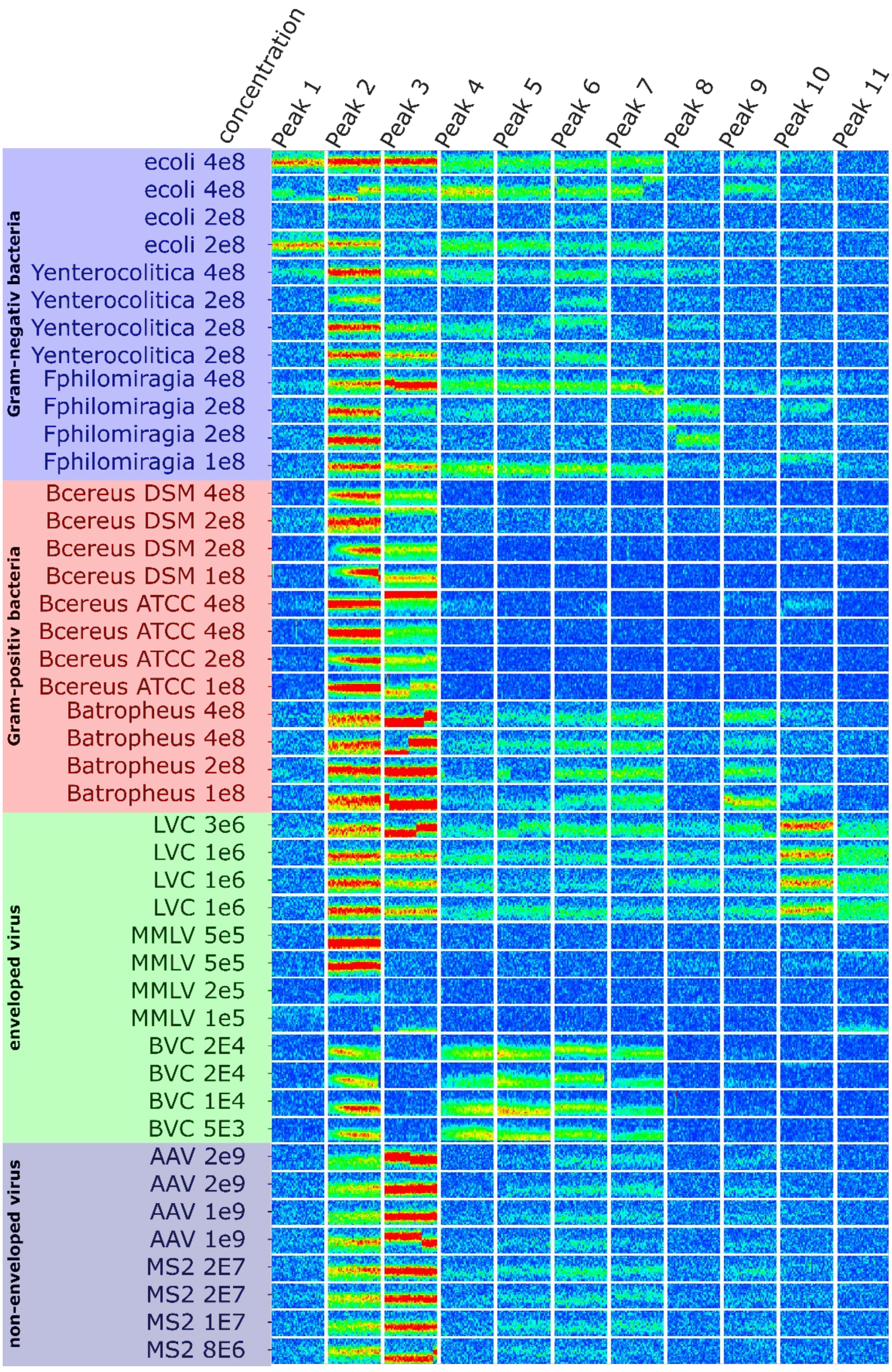


*Figure S16: Comparison of the 11 selected negative peaks (features) with the greatest variation in intensity.*

*Table S3: The weight before and after dipping the ferromagnetic vial into the fatty acids mixture of propionic acid, butyric acid and pentanoic acid with an average difference of 502 µg, a standard deviation of 109 µg and a resulting RSD of 21.74 %.*

| Measurement | Weight beforehand in g | Weight after dipping in g | Difference in weight in µg |
|---|---|---|---|
| 1 | 3.65556 | 3,65498 | 580 |
| 2 | 3,65061 | 3,65005 | 560 |
| 3 | 3,6511 | 3,65074 | 360 |
| 4 | 3,64995 | 3,64935 | 600 |
| 5 | 3,64197 | 3,64156 | 410 |

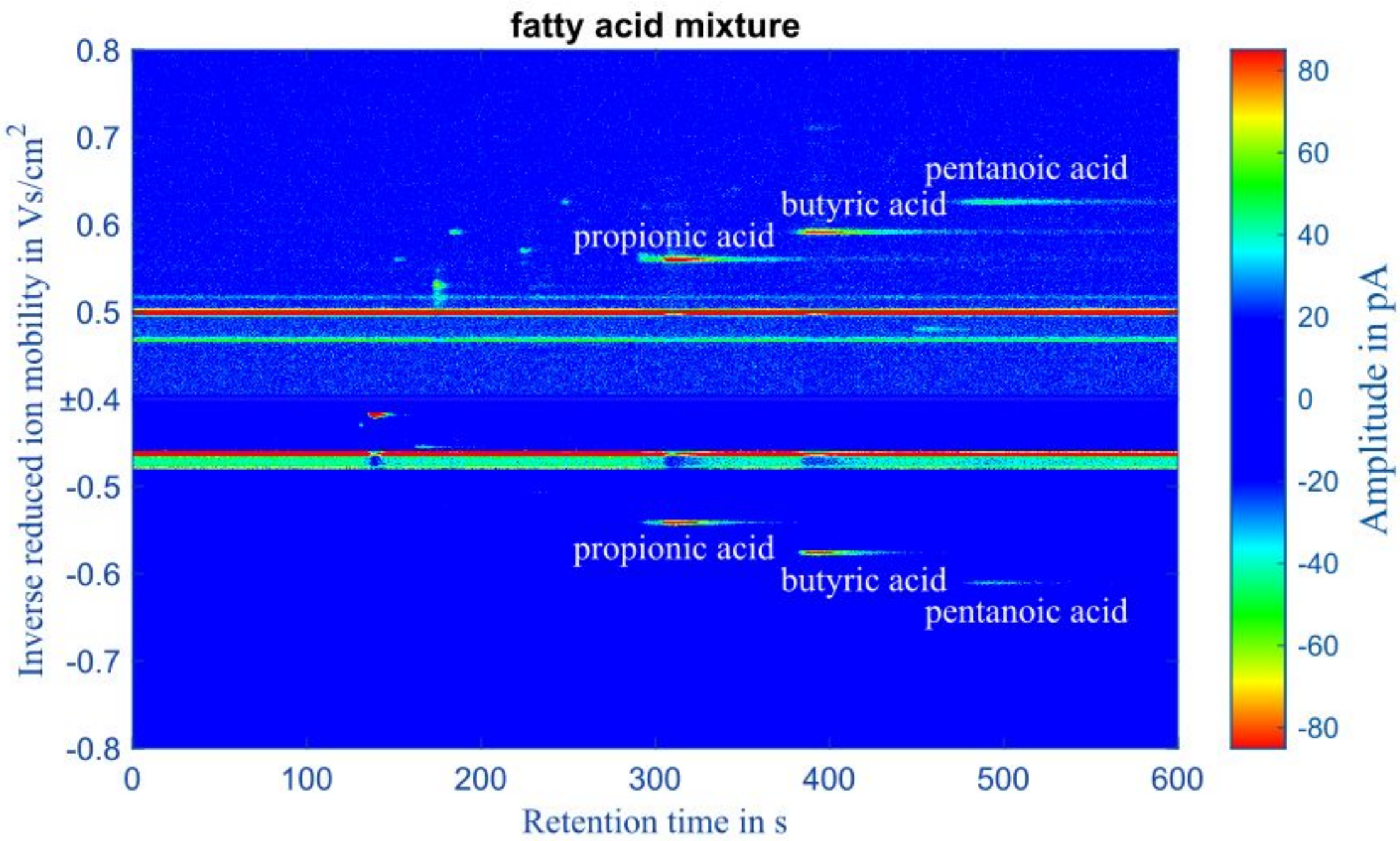


*Figure S17: 2D-chromatogram of the fatty acid mixture consisting of propionic acid, butyric acid, and pentanoic acid.*

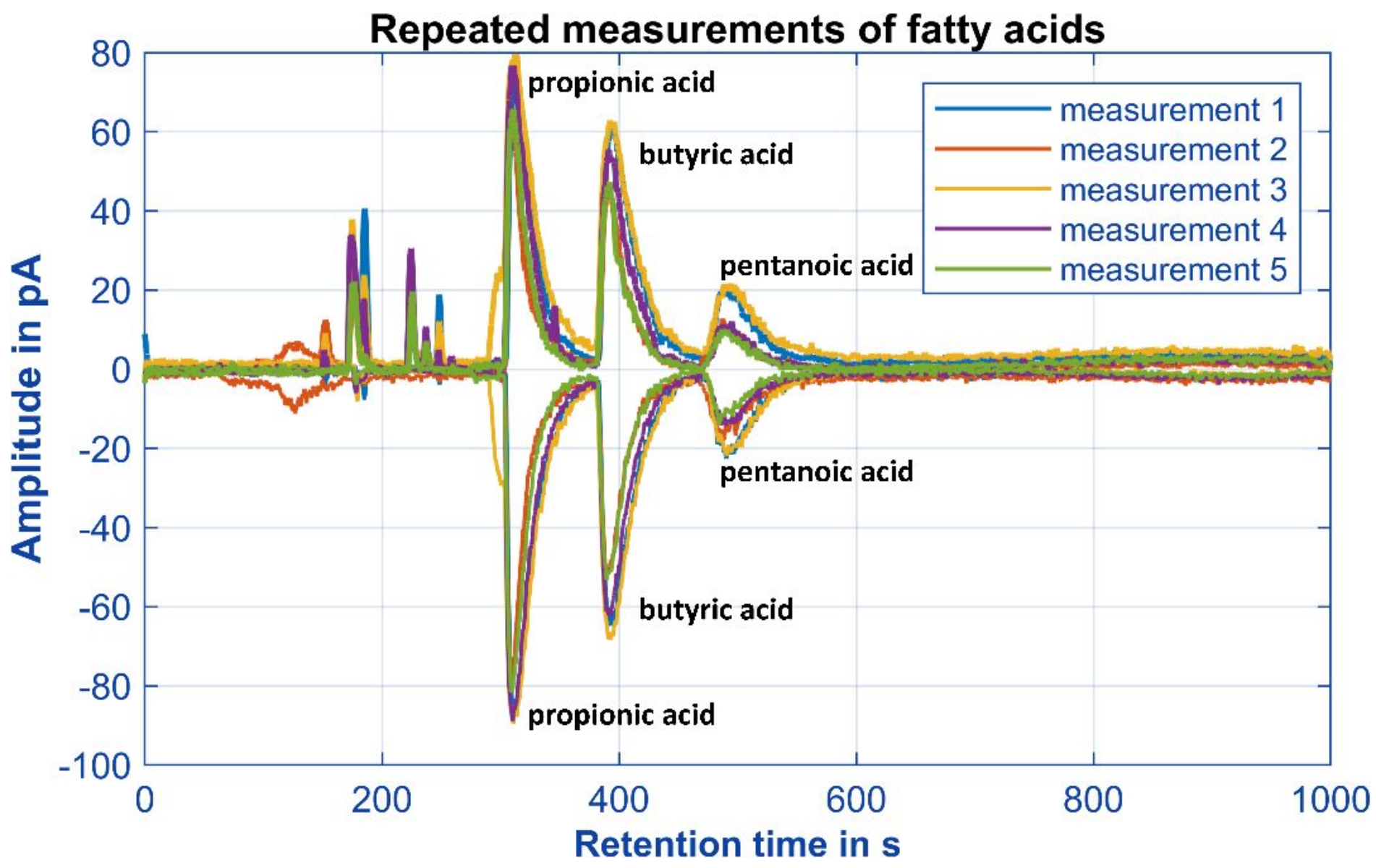


*Figure S18: Maximum amplitude of the 2D-chromatogram excluding the reactant ion peak over the retention time with 5 repeated measurements of fatty acids: propionic acid, butyric acid, and pentanoic acid.*

*Table S4: The average retention time, drift time, and amplitude with their respective standard deviation of the measured fatty acids.*

| Peaks | Retention time ± standard deviation in s | Reduced ion mobility ± standard deviation in ms | Amplitude ± standard deviation in pA |
|---|---|---|---|
| Positive peak 1 | 310.72±1.01 | 1.79±0.01 | 72.46±7.26 |
| Positive peak 2 | 392.15±0.74 | 1.70±0.01 | 54.58±7.55 |
| Positive peak 3 | 490.82±3.64 | 1.61±0.01 | 15.34±3.64 |
| Negative peak 1 | 310.38±0.71 | 1.86±0.01 | 84.55±5.14 |
| Negative peak 2 | 391.47±1.49 | 1.75±0.01 | 60.13±7.29 |
| Negative peak 3 | 488.71±2.78 | 1.65±0.01 | 18.18±4.03 |

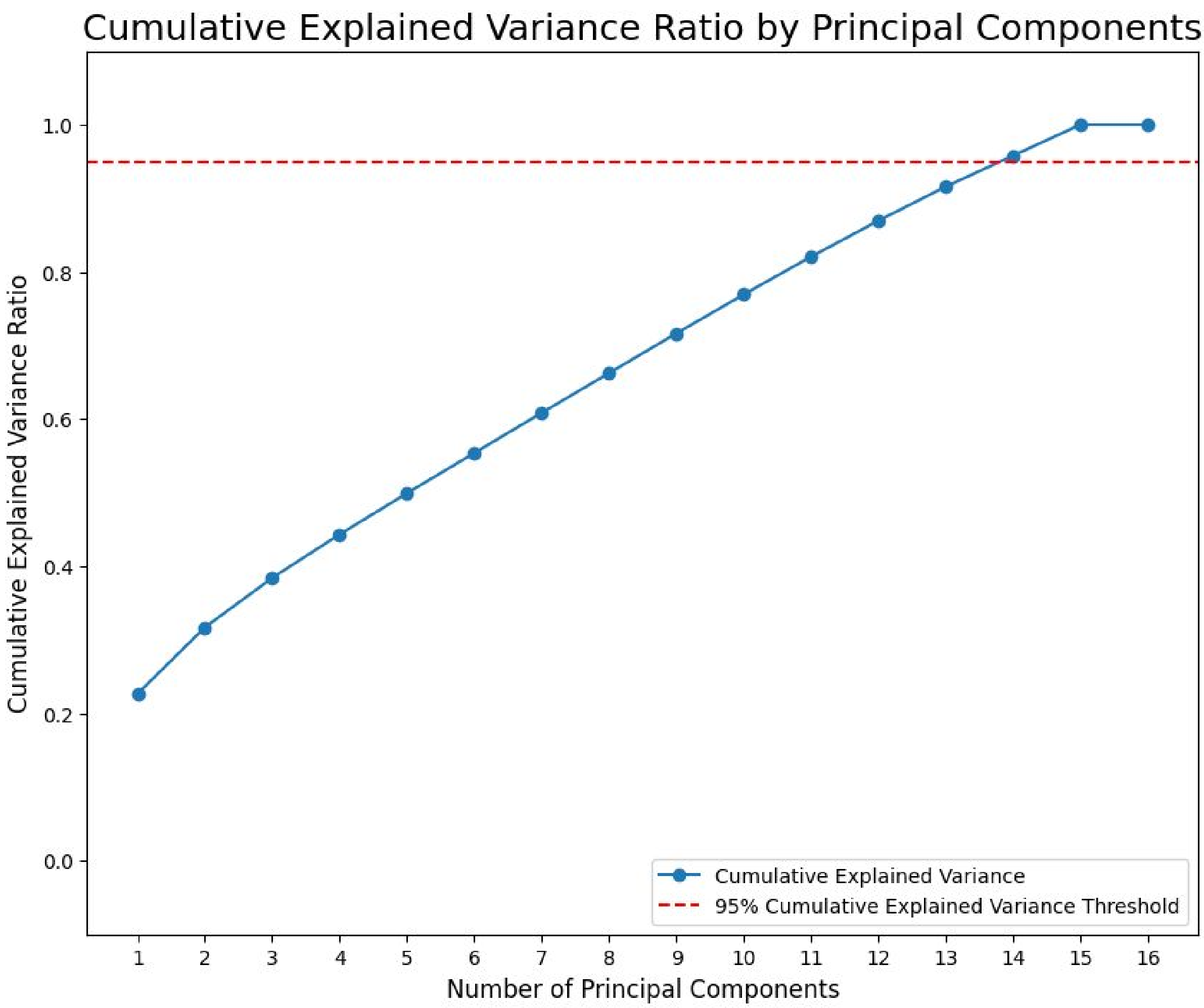


*Figure S19: Cumulative explained variance ratio by principal component analysis.*

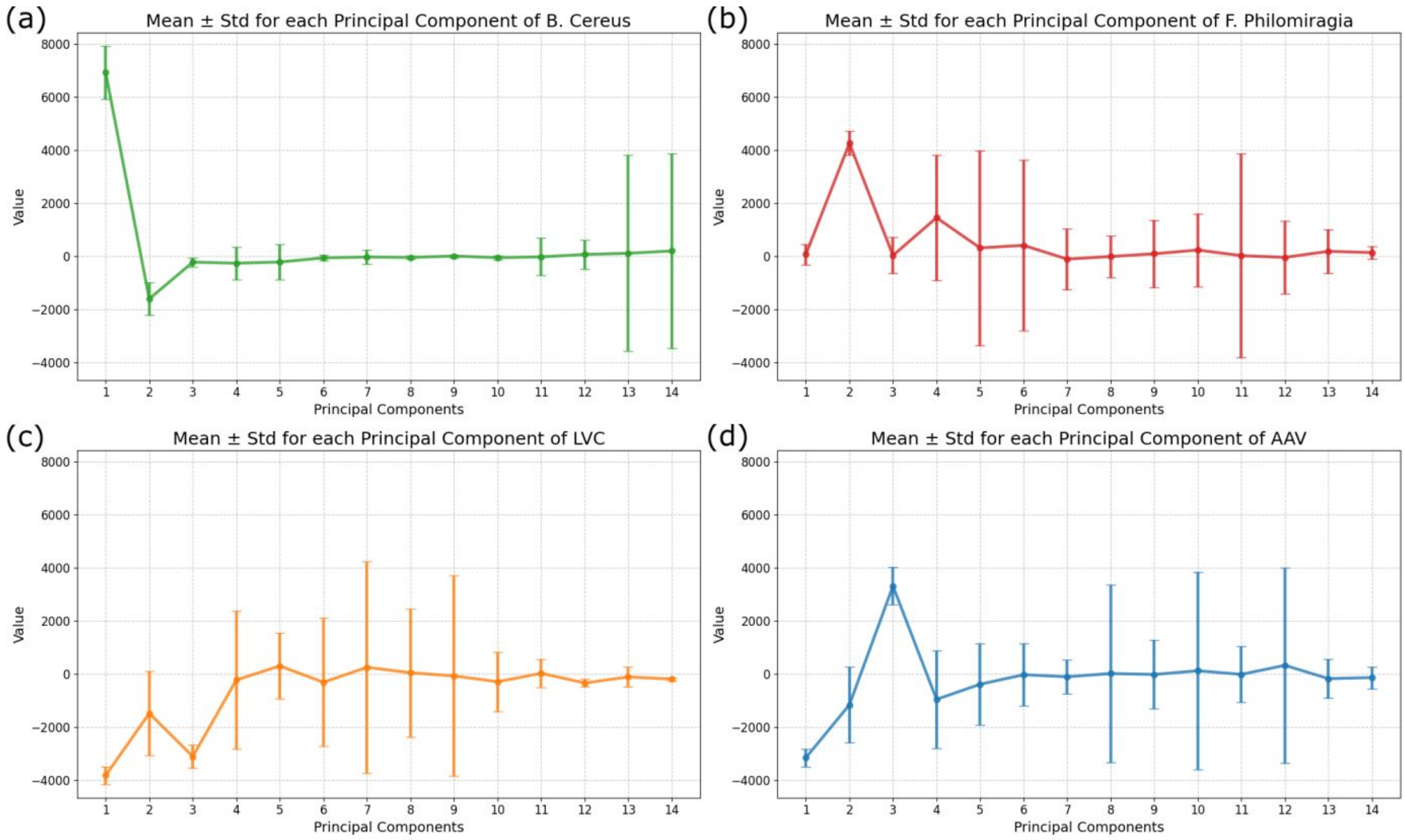


*Figure S20: Average and standard deviation of each principal component for each representative class ((a): B. cereus, (b): F. philomiragia, (c): LVC, (d): AAV).*

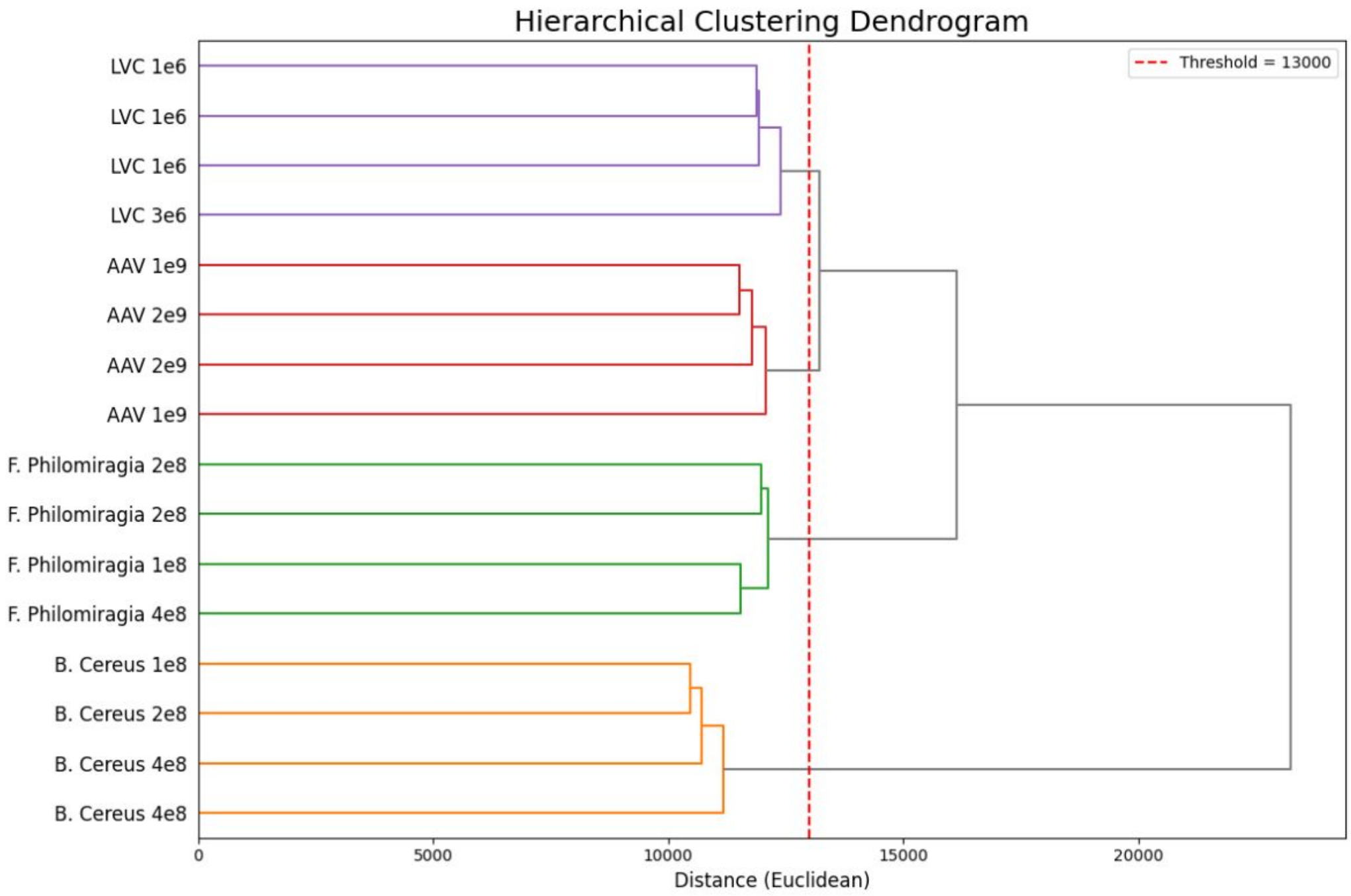


*Figure S21: Hierarchical clustering dendrogram of the representative dataset.*
